# Spatiotemporal evolution of early innate immune responses triggered by neural stem cell grafting

**DOI:** 10.1186/scrt147

**Published:** 2012-12-14

**Authors:** Kristien Reekmans, Nathalie De Vocht, Jelle Praet, Erik Fransen, Debbie Le Blon, Chloé Hoornaert, Jasmijn Daans, Herman Goossens, Annemie Van der Linden, Zwi Berneman, Peter Ponsaerts

**Affiliations:** 1 Laboratory of Experimental Hematology, Faculty of Medicine and Health Sciences, University of Antwerp, Universiteitsplein 1, Antwerp-Wilrijk, 2610, Belgium; 2 Vaccine and Infectious Disease Institute (Vaxinfectio), Faculty of Medicine and Health Sciences, University of Antwerp, Universiteitsplein 1, Antwerp-Wilrijk, 2610, Belgium; 3 BioImaging Laboratory, Faculty of Pharmaceutical, Biomedical and Veterinary Sciences, Department of Biomedical Sciences, Universiteitsplein 1, Antwerp-Wilrijk, 2610, Belgium; 4 StatUa Centre for Statistics, City Campus, University of Antwerp, Prinsstraat 13, Antwerp, 2000, Belgium

## Abstract

**Introduction:**

Transplantation of neural stem cells (NSCs) is increasingly suggested to become part of future therapeutic approaches to improve functional outcome of various central nervous system disorders. However, recently it has become clear that only a small fraction of grafted NSCs display long-term survival in the (injured) adult mouse brain. Given the clinical invasiveness of NSC grafting into brain tissue, profound characterisation and understanding of early post-transplantation events is imperative to claim safety and efficacy of cell-based interventions.

**Methods:**

Here, we applied *in vivo *bioluminescence imaging (BLI) and post-mortem quantitative histological analysis to determine the localisation and survival of grafted NSCs at early time points post-transplantation.

**Results:**

An initial dramatic cell loss (up to 80% of grafted cells) due to apoptosis could be observed within the first 24 hours post-implantation, coinciding with a highly hypoxic NSC graft environment. Subsequently, strong spatiotemporal microglial and astroglial cell responses were initiated, which stabilised by day 5 post-implantation and remained present during the whole observation period. Moreover, the increase in astrocyte density was associated with a high degree of astroglial scarring within and surrounding the graft site. During the two-week follow up in this study, the NSC graft site underwent extensive remodelling with NSC graft survival further declining to around 1% of the initial number of grafted cells.

**Conclusions:**

The present study quantitatively describes the early post-transplantation events following NSC grafting in the adult mouse brain and warrants that such intervention is directly associated with a high degree of cell loss, subsequently followed by strong glial cell responses.

## Introduction

Neural stem cells (NSCs) are defined as a population of self-renewing multipotent progenitor cells present in the developing and adult central nervous system (CNS) [1]. Despite their specific spatiotemporal occurrence *in vivo, ex vivo *culture expansion of NSCs derived from various sources (for example, embryonic or postnatal brain and embryonic stem cells) was shown to be relatively straightforward. Moreover, given the observation that *ex vivo *cultured neurosphere-derived NSCs and monolayer-cultured NSC populations can be triggered to differentiate into neurons, astrocytes and oligodendrocytes, it is hoped that NSCs, or more committed (progenitor) cells derived thereof, will become part of novel treatment options for a variety of CNS disorders. To date, several studies have reported the clinical benefit of NSCs following intraperitoneal, intravenous, intraventricular, intrathecal and intra-tissue grafting in various models of neuroinflammation, neurodegeneration or injury [1-4]. However, despite many suggestive literature reports, it still remains elusive whether the actual clinical benefit of grafted NSCs is due to cell integration, trophic support, immunomodulation or other yet to be defined mechanisms [5-7]. In this context, preceding work by others [8] and us [9] determined the actual survival of grafted NSCs after at least two weeks to be less then 2% upon direct grafting into healthy or inflamed brain tissue. In addition, our preceding histological analyses indicated the presence of both microglia and astrocytes within and surrounding the NSC graft site at two weeks post-implantation [9]. Following these observations, our next research aim was to determine whether the observed glial cell responses: (i) were directly executed against the grafted NSC (or their reporter proteins), or (ii) resulted as a consequence of immediate cell graft mortality. In order to investigate both hypotheses, the present study determined the following parameters at multiple early time points (that is, day 0, 1, 3, 5, 7 and 14) post-implantation: (i) the actual survival of grafted NSC, (ii) the occurrence of cellular hypoxia, and (iii) the occurrence and/or maintenance of cell graft-induced glial cell responses.

## Materials and methods

### Cell implantation experiments

NSC genetically engineered with the Luciferase and eGFP reporter proteins (NSC-Luc/eGFP, FVB-background) were cultured and characterised as previously described [10]. For cell implantation experiments, adult female Friend leukemia virus B (FVB)/NCrl mice (n = 33) were obtained from Charles River Laboratories (Wilmington, MA, USA - strain code 207). Cell implantation of NSC-Luc/eGFP (2.5 × 10^5 ^cells in 2 μl PBS) was reproducibly targeted to the right hemisphere at the following coordinates relative to bregma: 2 mm posterior, 2 mm lateral, and 2.25 mm ventral. All surgical interventions were performed under sterile conditions, as previously described [9-11]. For all experiments, mice were kept in a normal day-night cycle (12/12) with free access to food and water. All experimental procedures were approved by the Ethics Committee for Animal Experiments of the University of Antwerp (UA) (approval no. 2011/13).

### *In vivo *bioluminescence imaging

*In vivo *bioluminescence imaging (BLI) was performed at different time points post-implantation (day 1, 3, 5, 7, 10 and 14) using a real-time photon-imager system (Biospace, Centennial, CO, USA), according to previously optimised procedures [10-12]. Using the M3 Vision software (Biospace, Centennial, CO, USA), light emission was measured from a fixed region of interest on the mouse head, and values of signal intensity are presented as the average number of photons/s/sr/cm^2 ^over a 3-minute time period. An additional region of interest was drawn on the mouse shoulder and considered as background signal.

### Histological analysis - immunofluorescence staining

Before sacrifice (1.5 hours), mice used in this study were injected intraperitoneally with Hypoxyprobe-1 (HPI Inc, Burlington, MA, USA), according to the manufacturer's instructions. Preparation of brain tissue for histological examination was performed according to previously optimised procedures [9]. Serial 10-μm thick cryosections were obtained from the entire implant region using a Microm HM5000 cryostat (Prosan, Merelbeke, Belgium), consecutively marked and missing slides were noted. For further immunofluorescence analysis of tissue sections, antibody staining was performed as previously described [9,10,12], using the following antibodies: (i) a rabbit anti-mouse ionized calcium binding adaptor molecule 1 (Iba1) antibody (1/200) (Wako chemicals, Osaka, Japan; 019-19714) and (ii) a rabbit anti-pimonidazole (Hypoxyprobe-1) antibody (1/200) (HPI Inc., Burlington, MA, USA; Pab2627), all in combination with a secondary donkey anti-rabbit AlexaFluor 555 antibody (1/500) (Life Technologies, Carlsbad, CA, USA; A31572), (iv) a rabbit anti-mouse S100B antibody (1/200) (Abcam, Cambridge, UK; 52642) in combination with a secondary donkey anti-rabbit AlexaFluor 555 antibody (1/1000) (Life Technologies, Carlsbad, CA, USA; A31572), (v) a mouse anti-mouse GFAP antibody (1/400) (Millipore, Billerica, MA, USA; MAB360) in combination with a secondary goat anti-mouse AlexaFluor 555 (1/1000) (Life Technologies, Carlsbad, CA, USA; A21127), and (vi) a chicken anti-mouse MBP antibody (1/200) (Millipore, Billerica, MA, USA; AB9348) in combination with a secondary donkey anti-chicken DyLight 549 (Jackson Immunoresearch, Suffolk, UK; 703-506-155). The presence of terminal deoxynucleotidyl transferase dUTP nick end labelling (TUNEL)+ apoptotic cells was investigated using the *In Situ *Cell Death Detection Kit TMR Red (Roche, Penzberg, Germany; 12156792910), according to manufacturer's instructions. Nuclear staining was performed using a TOPRO3 deep red stain (1/200) (Life Technologies, Carlsbad, CA, USA).

### Histological analysis - quantitative analysis

Quantitative analysis of cell graft survival, glial cell responses and cellular hypoxia were performed using NIH ImageJ analysis software (ImageJ) and TissueQuest immunofluorescence analysis software (TissueGnostics GmbH, Vienna, Austria), allowing determination of the following parameters: (i) total graft site volume in mm^3^, (ii) density of eGFP^pos ^NSC-luc/eGFP within the graft site provided in number of cells/mm^3 ^(six data counts per cell graft analysed), (iii) cell graft survival provided in absolute numbers and as % calculated to the initial number of grafted cells, (iv) density of Iba1^pos ^microglia within the graft site provided in number of cells/mm^3 ^(three data counts per cell graft analysed), (v) density of Iba1^pos ^microglia within the implant border (that is, region extending 100 μm from the implant site) provided in number of cells/mm^3 ^(three data counts per cell graft analysed), (vi) density of S100B^pos ^astrocytes within the graft site in number of cells/mm^3 ^(three data counts per cell graft analysed), (vii) density of S100B^pos ^astrocytes within the implant border provided in number of cells/mm^3 ^(three data counts per cell graft analysed), (viii) the degree of glial fibrillary acidic protein (GFAP)^pos ^astrogliosis within the graft site provided as % astrogliosis (that is, image-covering of GFAP staining) (one data count per cell graft analysed), (ix) the degree of GFAP^pos ^astrogliosis within the implant border provided as % astrogliosis (one data count per cell graft analysed), (x) the percentage of Hypoxyprobe-1^pos ^eGFP^pos ^NSC versus total eGFP^pos ^NSC within the implant zone (one data count per cell graft analysed).

### Statistical analysis

All statistical analyses were performed using the statistical package R, version 2.13.1, with linear mixed models fitted using the lme function in the nlme package. Differences in graft site volume between day 0 and day 14 post-grafting were tested using the Mann-Withney *U*-test. The evolution over time of NSC graft survival was modelled using piecewise linear regression, taking the knot at day 1 post-implantation. Using this model, separate regression slopes up to day 1 and beyond day 1 (that is, from on day 3) were estimated and tested for significance. The evolution over time of the obtained *in vivo *bioluminescence signal intensities, microglia densities, astrocyte densities and the degree of astrogliosis was modelled using piecewise linear mixed model analysis. A random intercept for individual was added to the model to account for the dependence between observations from the same individual. Values for bioluminescence signal intensity, microglia densities and astrocyte densities were log-transformed to obtain a more normally distributed outcome variable. The position of the knot was determined based upon visual inspection of the data. Separate regression slopes before and after the knot were estimated and tested for significance. For all analyses, a *P*-value < 0.05 was considered statistically significant.

## Results

### Longitudinal *in vivo *bioluminescence imaging of neural stem cell grafts

**Figure 1:**
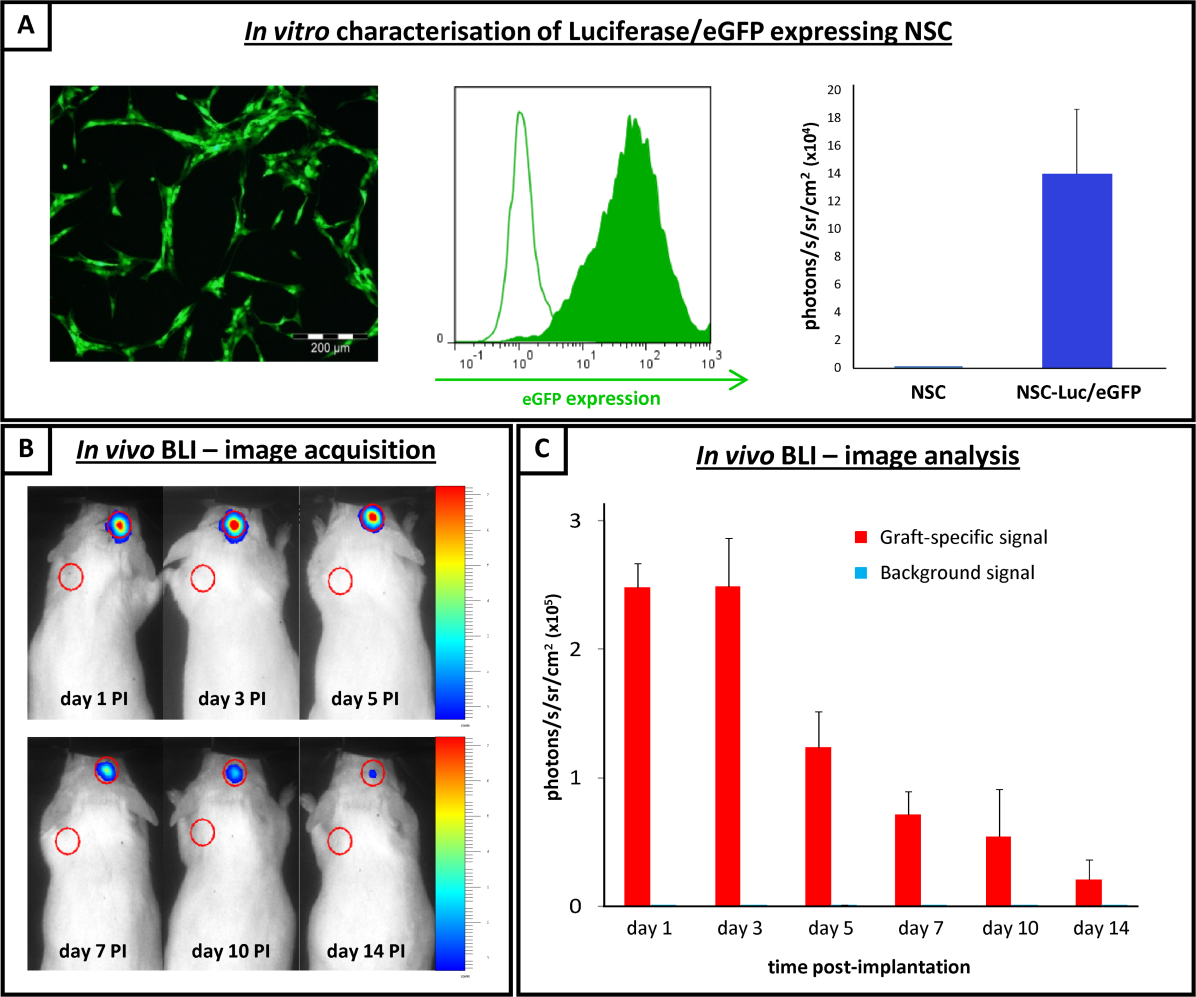
**Longitudinal *in vivo *bioluminescence imaging of neural stem cell-Luciferase/enhanced green fluorescent protein (NSC-Luc/eGFP) grafts**. (A) *In vitro *characterisation of NSC-Luc/eGFP. Left, representative fluorescence microscopy image of Luciferase/eGFP-expressing NSC (NSC-Luc/eGFP) used in this study. Green colour, direct eGFP fluorescence. Middle histogram overlay, representative flowcytometric analysis of parental NSC and NSC-Luc/eGFP. Open black histogram, control background fluorescence in FL-1 green channel from parental NSC. Filled green histogram, direct eGFP fluorescence in FL-1 green channel from NSC-Luc/eGFP. Right, *in vitro *bioluminescence analysis of 1 × 10^5 ^parental NSC and NSC-Luc/eGFP. Data are expressed as photons/s/sr/cm^2 ^from a 5-minute time period (± standard error of the mean (SEM), n = 4). (B) *In vivo *bioluminescence imaging (BLI) - image aquisition. Representative time course image showing *in vivo *BLI of mice grafted with 1.5 × 10^5 ^NSC-Luc/eGFP in the central nervous system (CNS). Images were acquired at day 1 (n = 25), day 3 (n = 15), day 5 (n = 15), day 7 (n = 10), day 10 (n = 5) and day 14 (n = 5) post-implantation. Regions of interest are drawn on the mouse head, where NSC-Luc/eGFP were injected, and on the mouse shoulder, considered as background signal. A representative time course image was chosen out of five mice imaged for the whole time course. (C) *In vivo *BLI- image analysis. Quantitative analysis of *in vivo *BLI analysis at day 1, 3, 5, 7, 10 and 14 post-implantation. BLI signals (in photons/s/sr/cm^2^) are provided as the mean (± SEM) for all mice analysed, both from the specific region of interest on the mouse head (red bars) and from the control region of interest on the mouse shoulder (blue bars). Significant differences are described in the results section.

For cell grafting experiments, we used a previously engineered NSC line expressing both the Luciferase and eGFP reporter proteins (Figure 1A), further named as NSC-Luc/eGFP [10]. Following grafting of 1.5 × 10^5 ^NSC-Luc/eGFP in the CNS of immune-competent FVB mice (n = 33), longitudinal *in vivo *BLI was performed at day 1 (n = 25), day 3 (n = 15), day 5 (n = 15), day 7 (n = 10), day 10 (n = 5) and day 14 (n = 5) post-implantation. For quantitative analysis of the observed BLI signals, the mean cell graft-specific BLI signals from fixed regions on top of the mouse head (Figure 1B) and the mean background BLI signals from fixed control regions on the mouse shoulder (Figure 1B) were plotted versus time post-implantation (Figure 1C). Regression analysis of the data indicates a significant decrease in BLI signal beyond day 3 post-implantation (*P *< 0.0001), indicative of a progressive decrease of cell viability within NSC-Luc/eGFP grafts.

### Quantitative analysis of neural stem cell graft survival

**Figure 2:**
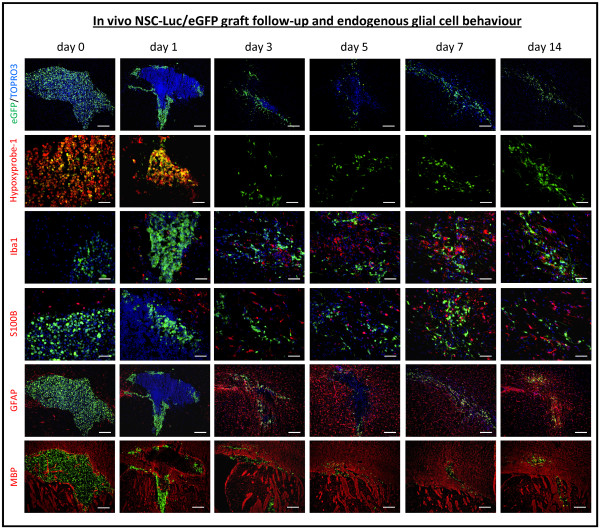
**Histological analysis of neural stem cell (NSC) graft survival and endogenous glial cell responses**. First row, NSC-Luciferase/enhanced fluorescent green protein (Luc/eGFP) graft survival. Direct eGFP fluorescence (green) combined with TOPRO3 staining (false colour representation in blue) at day 0, 1, 3, 5, 7 and 14 post-implantation. Representative images were chosen from multiple stained slides (n = 6 to 9 for eGFP/TOPRO3 combination) per mouse analysed at each time point. The provided scale bars indicate 200 μm. Second row, cellular hypoxia. Direct eGFP fluorescence (green) combined with Hypoxyprobe-1staining (red) at day 0, 1, 3, 5, 7 and 14 post-implantation. Representative images were chosen from two to five mice analysed at each time point. The provided scale bars indicate 50 μm. Third, fourth and fifth row, endogenous glial cell behaviour. Direct eGFP fluorescence (green) combined with TOPRO3 staining (false colour representation in blue) and combined with immunofluorescence staining for ionized calcium binding adaptor molecule 1 (Iba1) (red, fourth row), S100 calcium binding protein B (S100B) (red, fifth row) or glial fibrillary acidic protein (GFAP) (red, sixth row) at day 0, 1, 3, 5, 7 and 14 post-implantation. Representative images were chosen from multiple stained slides (n = 3 for eGFP/TOPRO3/Iba1 combination, n = 3 for eGFP/TOPRO3/S100B combination and n = 1 for eGFP/TOPRO3/GFAP) per mouse analysed at each time point (n = 4/5). The provided scale bars indicate 50 μm for Iba1 and S100B images and 200 μm for GFAP images. Sixth row, graft site remodelling. Direct eGFP fluorescence (green) combined with myelin base protein (MBP) staining (red) at day 0, 1, 3, 5, 7 and 14 post-implantation. Representative images were chosen from multiple mice analysed at each time point (n = 2). The provided scale bars indicate 200 μm.

**Figure 3:**
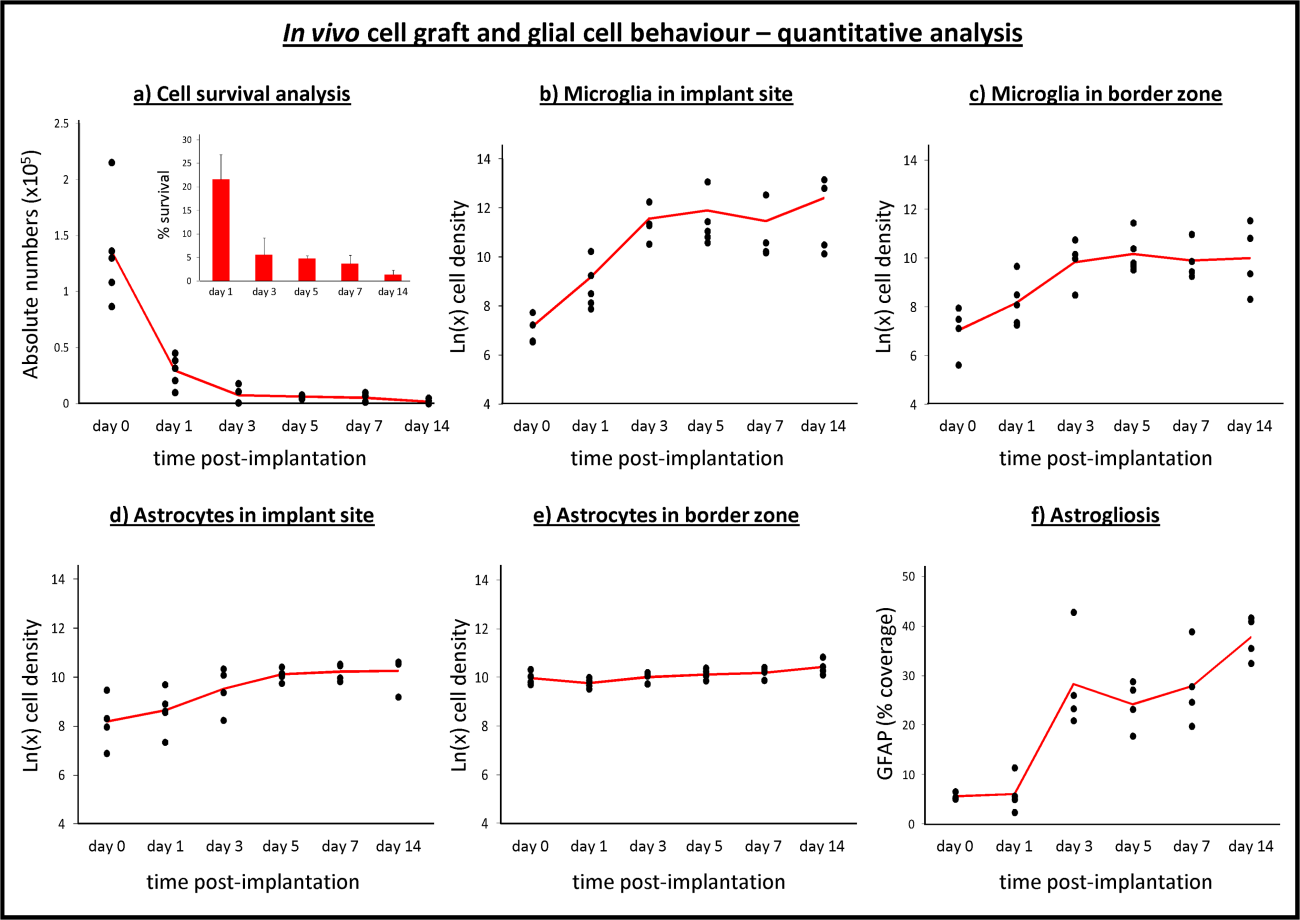
***In vivo *cell graft and glial cell behaviour - quantitative analysis**. (a) Survival of grafted neural stem cell-Luciferase/enhanced fluorescent green protein (NSC-Luc/eGFP). Presented data are the estimated total number of eGFP-expressing NSC-Luc/eGFP detected within the implant site for each mouse analysed (n = 5 for day 0, n = 5 for day 1, n = 4 for day 3, n = 4 for day 5, n = 4 for day 7 and n = 4 for day 14). The red line indicates the average total number of eGFP-expressing NSC-Luc/eGFP at each time point analysed. The inset graph represents the mean % (± standard error of the mean (SEM)) of NSC-Luc/eGFP graft survival at each time point analysed from day 1 post-implantation. Significant differences are described in the results section. (b-e) Cellular density of ionized calcium binding adaptor molecule 1 (Iba1)^pos ^microglia and S100 calcium binding protein B (S100B)^pos ^astrocytes within the implant site and within the implant border. Presented data are the ln(x)-transformed values of estimated total density of microglia and astrocytes within the implant border for each mouse analysed (n = 4 for day 0, n = 5 for day 1, n = 4 for day 3, n = 5 for day 5, n = 4 for day 7 and n = 4 for day 14). The red line indicates the average total cellular density at each time point analysed. Significant differences are described in the results section. (f) Astrogliosis within the implant site and implant border (both areas are combined here). Presented data indicate the average degree of glial fibrillary acidic protein (GFAP)^pos ^astrogliosis (based on image coverage after GFAP staining) for each mouse analyses (n = 4 for day 0, n = 5 for day 1, n = 4 for day 3, n = 5 for day 5, n = 4 for day 7 and n = 4 for day 14). The red line indicates the average degree of astrogliosis at each time point analysed. Significant differences are described in the results section.

Histological analyses of brain tissue from cell-grafted mice were performed at day 0 (4 hours post-implantation, n = 5), day 1 (n = 5), day 3 (n = 4), day 5 (n = 4), day 7 (n = 4) and day 14 (n = 4) post-implantation. For this, cryosections were prepared from the whole graft site area and screened for the presence of eGFP-expressing NSC-Luc/eGFP implants. Representative histological images of NSC-Luc/eGFP implants, provided in Figure 2 (first row), already indicate extensive cell death at day 1 post-implantation. The latter is clearly visualised by the loss of eGFP expression within NSC-Luc/eGFP grafts at day 1 as compared to the uniform eGFP expression within NSC-Luc/eGFP grafts at day 0. From day 7 post-implantation, the necrotic core of NSC-Luc/eGFP implants has disappeared, while remaining NSC-Luc/eGFP have dispersed along the white matter tracts of the capsula externa and corpus callosum. Larger images of those presented in Figure 2 are provided in Figure S1 in Additional file 1. Further quantitative analysis estimated the actual number of grafted NSC-Luc/eGFP at day 0 post-implantation and the number of surviving NSC-Luc/eGFP at day 1, day 3, day 5, day 7 and day 14 post-implantation (Figure 3a). Presented data indicate a dramatic initial cell loss (up to 80%) within the first 24 hours post-implantation (*P *< 0.0001), which slowly continues between day 1 and 14 post-implantation (*P *= 0.004). Based on the number of grafted cells at day 0, graft survival was estimated to be 22%, 6%, 5%, 4% and 1% respectively, at day 1, day 3, day 5, day 7 and day 14 post-implantation (Figure 3a inset).

### Excessive apoptotic cell loss coincides with cellular hypoxia within NSC grafts

**Figure 4:**
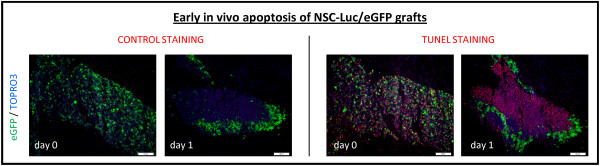
**Histological analysis of *in vivo *neural stem cell (NSC) graft apoptosis**. Direct enhanced fluorescent green protein (eGFP) fluorescence (green) combined with TOPRO3 staining (false colour representation in blue) and combined with the control immunofluorescence staining for terminal deoxynucleotidyl transferase dUTP nick end labeling (TUNEL) (red, CONTROL STAINING) and the specific immunofluorescence staining for TUNEL+ apoptotic cells (red, TUNEL STAINING) at day 0 and 1 post-implantation. Representative images were chosen from multiple mice analysed at each time point (n = 2). The provided scale bars indicate 100 μm.

As shown by the representative images in Figure 2 (second row), it is clear at day 0 and day 1 post-grafting that the majority of grafted NSC-Luc/eGFP are under hypoxic condition as demonstrated by immunostaining of tissue sections for Hypoxyprobe-1 (an *in vivo *probe for labelling of hypoxic cells). Further quantitative image analysis indeed confirmed a high percentage of hypoxic cells among (still) viable eGFP expressing NSC-Luc/eGFP at day 0 (mean 74.3% ± standard deviation (SD) 8.3%) and day 1 (54.9% ± 5.5%) post-grafting, which was not detected at later time points post-grafting in the few surviving NSC-Luc/eGFP. Moreover, as shown in Figure 4, already at 4 hours post-grafting (day 0) TUNEL reactivity can be observed within the population of grafted NSC-Luc/eGFP. In contrast, at day 1 post-grafting, TUNEL reactivity is highly apparent within the eGFP-negative necrotic core of the NSC-Luc/eGFP graft, but not in the few surviving NSC-Luc/eGFP at the border of the graft. Larger images of those presented in Figure 4 are provided in Figure S2 in Additional file 1. Based on these data, we suggest that excessive cell loss observed within the first 24 hours post-grafting might be (partially) initiated by cellular hypoxia (and presumably also lack of nutrients) within the core of NSC-Luc/eGFP grafts, resulting in massive apoptotic cell death.

### Quantitative analysis of glial cell responses following neural stem cell grafting

Since around 95% of cell graft mortality can be observed within the first 48 hours following NSC-Luc/eGFP grafting, glial cell responses are inevitable evoked. Therefore, we investigated the temporal behaviour of microglia and astrocytes following NSC-Luc/eGFP grafting. From the representative data provided in Figure 2 (third row) no direct microglial response can be observed at day 0 post-implantation, as indicated by the absence of staining for Iba1^pos ^microglia. However, on day 1 post-implantation, and coinciding with the presence of a large number of necrotic cells, an accumulation of Iba1^pos ^microglia within and surrounding the graft site can be observed, which remains detectable from day 3 post-implantation. Next, although the number of S100B^pos ^astrocytes does not appear to change largely over time (Figure 2, fourth row), a high degree of astrogliosis, based on staining for GFAP (Figure 2, fifth row), is apparent at from day 3 post-implantation. Further quantitative image analysis of glial cell responses evoked by grafted NSC/Luc-eGFP focused on: (a) the implant site, delineated based on eGFP expression, and (b) the implant border, delineated as a 100-μm border around the implant site. Regarding microglial cell recruitment (Figure 3b and 3c), Iba1^pos ^microglia density significantly increases until day 3 post-implantation within and surrounding the graft site (for both *P *< 0.0001). However, no further increase (or decrease) in microglia density was observed within or surrounding the graft site beyond day 3 post-implantation (respectively *P *= 0.76 and *P *= 0.96). Regarding astroglial cell recruitment (Figure 3d and 3e), S100B^pos ^astrocyte density significantly increased within the graft site until day 5 post-grafting (*P *< 0.0001), with no increase beyond day 5 (*P *= 0.27). Within the implant border, astrocyte density slightly increased between day 0 and day 14 post-grafting (*P *= 0.0021). All absolute microglia and astrocyte cell densities within and surrounding the graft site at day 0, 1, 3, 5, 7 and 14 are provided in Table 1. Although the number of astrocytes recruited within and surrounding the graft site is less impressive compared to microglia recruitment, staining for GFAP was performed in order to determine GFAP^pos ^astroglial scarring within and surrounding the graft site. Presented data (Figure 3f) clearly indicate that the degree of GFAP^pos ^astrogliosis within and surrounding the graft site (both areas combined) significantly increases throughout the whole observation period, with the highest increase occurring prior to day 3 post-implantation (*P *< 0.0001), and a reduced, although significant (*P *= 0.006), increase beyond day 3 post-implantation.

**Table 1:** Astroglial and microglial densities following intracerebral implantation of NSC-Luc/eGFP.

	Time post-implantation					
	
	Day 0	Day 1	Day 3	Day 5	Day 7	Day 14
**Implant site**						
Microglia density	1,293 ± 768	10,017 ± 10,678	104,712 ± 75555	146,551 ± 188,791	94,803 ± 126,268	24,017 ± 250,036
Astrocyte density	5,382 ± 5,483	7,376 ± 5,653	18,059 ± 12,436	25,515 ± 5,986	28,900 ± 9,807	32,647 ± 15,108
**Border zone**						
Microglia density	1,537 ± 1,071	5,367 ± 5,951	24,551 ± 17,042	34,467 ± 33,349	25,062 ± 22,264	41,693 ± 44,628
Astroglial density	22,010 ± 6,202	17,757 ± 3,149	22,798 ± 4,216	25,308 ± 4,943	27,262 ± 5,647	34,603 ± 11,392

Absolute microglia and astrocyte cell densities (in number of cells/mm^3 ^± SD) within the implant site and the implant border at day 0, day 1, day 3, day 5, day 7 and day 14 post-grafting.

### Disruption of CNS architecture following neural stem cell transplantation

Inevitably, cell grafting in the CNS will induce damage to the CNS architecture due to needle insertion and the subsequent injection of a cell suspension. As shown by the representative images in Figure 2 (sixth row), it is clear that both the CNS architecture, as visualised by staining for myelin basic protein (MBP), and the NSC-Luc/eGFP graft site, as visualised by the presence of eGFP-expressing cells, undergo significant changes at early stages post-grafting. While initially grafted NSC-Luc/eGFP are clustered together and myelin conformation of the capsula externa is temporally spread out, this gradually remodels as the limited number of surviving NSC-Luc/eGFP spread along the capsula externa/corpus callosum, and the disturbance in myelin conformation partially restores itself. The latter is accompanied with a reduction of graft site volume (that is, the area in which eGFP-expressing cells can be found) from 0.35 ± (SD) 0.09 mm^3 ^at day 0 post-grafting to 0.07 ± 0.07 mm^3 ^at day 14 post-grafting (*P *= 0.015).

## Discussion

Over the past years, neural stem cell transplantation has been recognized as a promising novel therapeutic tool to treat CNS disorders for which today, no effective therapies are available. However, successful functional integration of grafted NSCs (or *in vitro*/*in vivo *NSC-derived cell types) is one of the major challenges in current neuroscience research, and if successful, will have an enormous impact on the future development of cell replacement therapies for CNS disorders. In order to contribute to this understanding, we have investigated the spatiotemporal evolution of NSC graft behaviour and endogenous glial cell responses at early time points post-implantation.

With regard to direct grafting of NSCs in CNS tissue, our results demonstrate that this procedure immediately results in: (i) significant disruption of the CNS architecture (Figure 2, sixth row) and (ii) substantial cell graft mortality (Figure 3a). First, the observed disruption of CNS architecture (although partially restored over time) will remain inevitable as long as cells (or cell-seeded scaffolds) are directly implanted into CNS tissue. Although alternative administration routes (that is, intraventricular, intrathecal or intravenous) have been suggested to facilitate cell delivery into injured CNS tissue, the cell number arriving at the target site is generally nihil to a few representative cells detected by histological analysis [13-15]. Second, apoptotic cell death within the first 24 hours post-grafting (Figure 4) can most likely be explained by the lack of oxygen (Figure 2, second row) and/or nutrients delivered to the NSC graft, which, in fact, is introduced without any structural and/or functional support. More recently, novel biomimetic strategies are emerging aiming to create a permissive micro environmental niche, thereby enhancing survival, differentiation and integration of grafted NSCs [16-18]. The latter will be inevitable, as a substantial amount of grafted cells need to survive following transplantation in order to functionally participate in neuronal circuitries. Of note, while we used both *in vivo *BLI (Figure 1c) and post-mortem histological analysis (Figure 3a) to determine cell graft survival, it should be noted that the observed *in vivo *BLI signals at day 1 and day 3 post-grafting are resulting from respectively 20% and 5% of the original number of grafted cells. Although we and others have associated this early *in vivo *BLI signal with complete cell graft survival, our results now clearly indicate that precaution has to be taken in the interpretation of early *in vivo *BLI data [10,11]. Nevertheless, *in vivo *BLI remains an interesting tool for longitudinal assessment of cell graft survival, as progressive cell loss of implanted NSC could clearly be demonstrated from on day 3 post-implantation (Figure 1c).

With regard to the observed glial cell responses, these are not surprising as the majority (up to 95%) of grafted NSCs already die within the first 24 to 48 hours post-grafting (Figure 3a). However, in our preceding studies regarding autologous and allogeneic stem cell grafting in the CNS of immune competent mice, we suggested that the occurrence of glial cell responses was most likely due to the recognition of the cellular implant itself, whereby autologous reporter-gene modified cellular grafts were immune-tolerated and allogeneic cellular grafts were immune-rejected. However, our early studies did not include full quantitative analysis of *in vivo *BLI and histology data. Based on our new data showing that NSC grafts already display a high degree of mortality before the initiation of glial cell responses (Figure 3b-f), the observed glial cell responses are more likely to be associated with phagocytocis of cellular debris rather than active recognition of grafted autologous NSC. Nevertheless, based on our preceding data regarding full rejection of allogeneic (but not autologous) cellular grafts, active distinguishment of autologous and allogeneic cellular grafts by microglia (or astrocytes) will thus most likely be occurring during or after the initial clearance of cellular debris. Following initial activation of glial cell responses, survival of grafted autologous NSC further declines to around 1% by week 2 post-implantation. Moreover, although endogenous microglia and astrocytes are able to produce either pro-inflammatory/neurotoxic or anti-inflammatory/neuroprotective factors depending on the type of activation [19,20], it is highly likely that the evoked immune responses following NSC implantation create an inhibitory pro-inflammatory environment, thereby further limiting the long-term survival of transplanted NSC [21]. Of note, additional immunophenotypical stainings were performed to determine the *in vivo *differentiation potential of the few surviving NSC-Luc/eGFP (data not shown). Results indicated that grafted NSC-Luc/eGFP did not change phenotypic properties between day 0 and day 14 post-grafting, that is, They remained SOX2^pos ^(as NSC marker), S100B^neg ^(as astrocyte marker), NeuN^neg ^(as neuronal marker) and CC1^neg ^(as oligodendrocytes marker). Likewise, proper *in vivo *differentiation and functional integration of the few surviving NSC is most likely inhibited by an endogenous glial-cell-induced pro-inflammatory immune environment [22-24].

## Conclusions

The present study quantitatively describes the early post-transplantation events following NSC grafting in the adult mouse brain and warrants that such intervention is associated with an immediate high degree of cell loss. The latter is subsequently followed by strong glial cell responses, presumably creating a non-permissive environment, which limit proper migration, differentiation and integration of grafted NSC into the host tissue. Unfortunately, these observations are generally not considered when evaluating the potential of pre-clinical cell therapy studies, as most reports lack detailed cell graft survival and/or glial reactivity analysis at early and late time-points post-grafting. Moreover, it is important to note that early NSC graft mortality and subsequent glial cell responses themselves might be responsible for many of the observed beneficial effects following cell transplantation in CNS disorders [2]. We therefore underscore the current need for a profound characterisation of all cellular and/or molecular interactions following cell grafting in the CNS, as the full potential of NSC transplantation therapy can only be determined following better understanding (and manipulation) of NSC graft survival and inhibitory immune responses.

## Abbreviations

BLI: bioluminescence imaging; CC1: anti-APC (Adenomatous polyposis coli protein) antibody; CNS: central nervous system; eGFP: enhanced green fluorescent protein; GFAP: glial fibrillary acidic protein; Iba-1: ionized calcium binding adaptor molecule 1; Luc: Luciferase; MBP: myelin basic protein; NeuN: neuronal nuclei; NSC: neural stem cell; PBS: phosphate-buffered saline; S100B: S100 calcium binding protein B; SOX2: sex-determining region Y-box 2; TUNEL: terminal deoxynucleotidyl transferase dUTP nick end labeling.

## Competing interests

The authors declare that they have no competing interests.

## Authors' contributions

KR, NDV, JP, HG, AvDL, ZB and PP designed the research study; KR, NDV, JP, DLB, CH and JD performed the research; EF performed the statistical analyses; KR and PP wrote the manuscript. All authors approved the final version of the manuscript.

## Supplementary Material

Additional file 1 **Histological analysis of neural stem cell (NSC) graft survival, endogenous glial cell responses and *in vivo *NSC graft apoptosis**. This file contains larger images of those presented in Figure 2 (Figure S1) and Figure 4 (Figure S2). Click here for file
