# Supplementary material for: Spatiotemporal evolution of early innate immune responses triggered by neural stem cell grafting
Source: Stem Cell Res Ther. 2012 Dec 14;3(6):56. doi: 10.1186/scrt147 (PMC3580486; doi:10.1186/scrt147)

Spatiotemporal evolution of early innate immune responses triggered by neural stem cell grafting.

Kristien Reekmans, Nathalie De Vocht, Jelle Praet, Erik Fransen, Debbie Le Blon, Chloé Hoornaert, Jasmijn Daans, Herman Goossens, Annemie Van der Linden, Zwi Berneman and Peter Ponsaerts.

Online Additional File 1

Figures S1:

Histological analysis of NSC graft survival and endogenous glial cell responses.

This section contains larger versions of the images presented in figure 2.

Images 1-6: NSC-Luc/eGFP graft survival. Direct eGFP fluorescence (in green) combined with TOPRO3 staining (false colour representation in blue) at day 0, day 1, day 3, day 5, day 7 and day 14 post-implantation. Representative images were chosen from multiple stained slides (n = 6-9 for eGFP/TOPRO3 combination) per mouse analysed at each time point. The provided scale bars indicate 200 μm. Images 7-12: cellular hypoxia. Direct eGFP fluorescence (in green) combined with Hypoxyprobe-1staining (red) at day 0, day 1, day 3, day 5, day 7 and day 14 post-implantation. Representative images were chosen from 2-5 mice analysed at each time point. The provided scale bars indicate 50μm. Images 13-18, 19-24, 25-30: endogenous glial cell behaviour. Direct eGFP fluorescence (in green) combined with TOPRO3 staining (false colour representation in blue) and combined with immunofluorescence staining for Iba1 (in red), S100B (in red) or GFAP (in red) at day 0, day 1, day 3, day 5, day 7 and day 14 post-implantation. Representative images were chosen from multiple stained slides (n = 3 for eGFP/TOPRO3/Iba1 combination, n=3 for eGFP/TOPRO3/S100B combination and n = 1 for eGFP/TOPRO3/GFAP) per mouse analysed at each time point (n = 4/5). The provided scale bars indicate 50 μm for Iba1 and S100B images and 200 μm for GFAP images. Images 31-36: graft site remodelling. Direct eGFP fluorescence (in green) combined with MBP staining (in red) at day 0, day 1, day 3, day 5, day 7 and day 14 post-implantation. Representative images were chosen from multiple mice analysed at each time point (n=2). The provided scale bars indicate 200μm.

eGFP/TOPRO3 – day 0

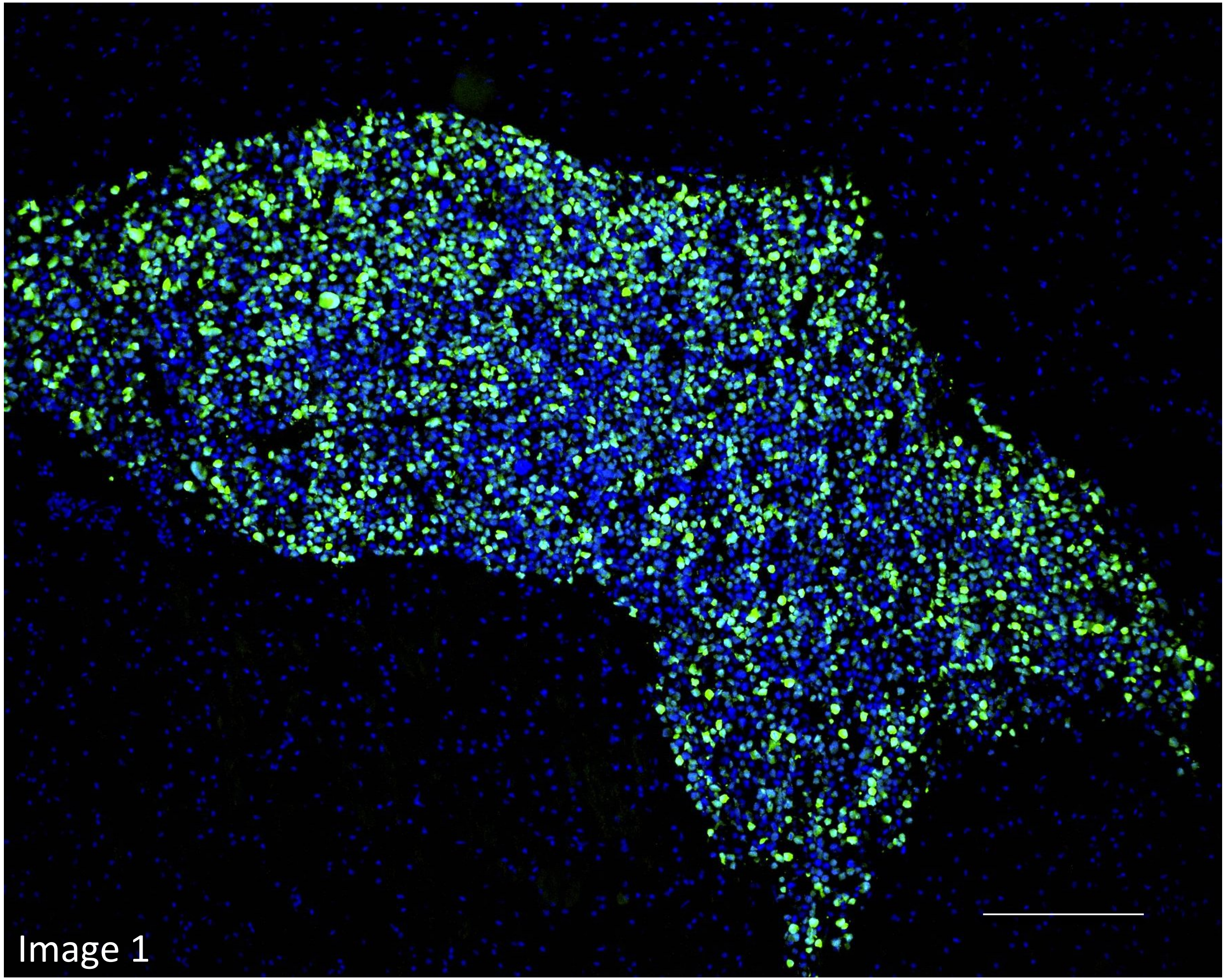

eGFP/TOPRO3 – day 1

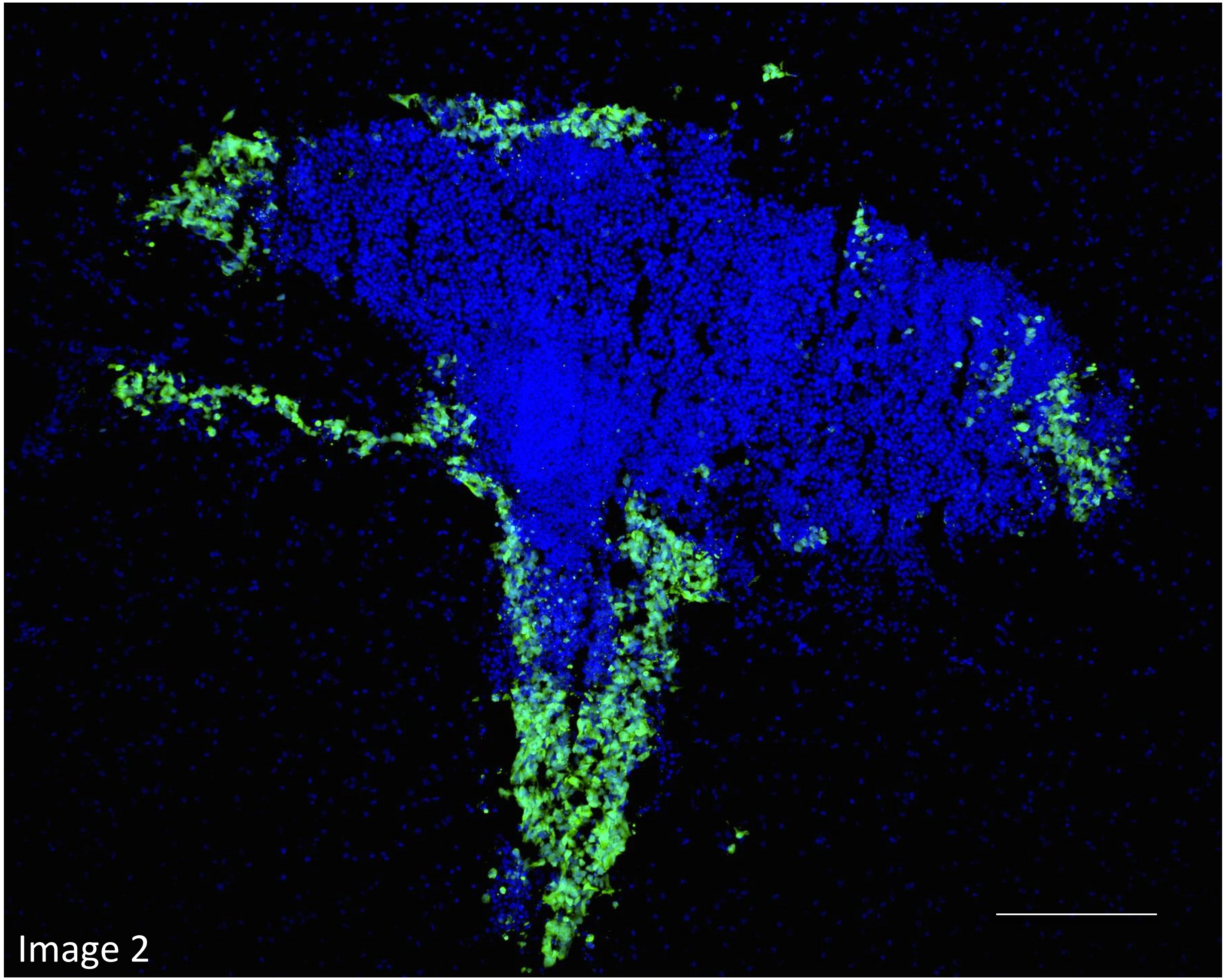

eGFP/TOPRO3 – day 3

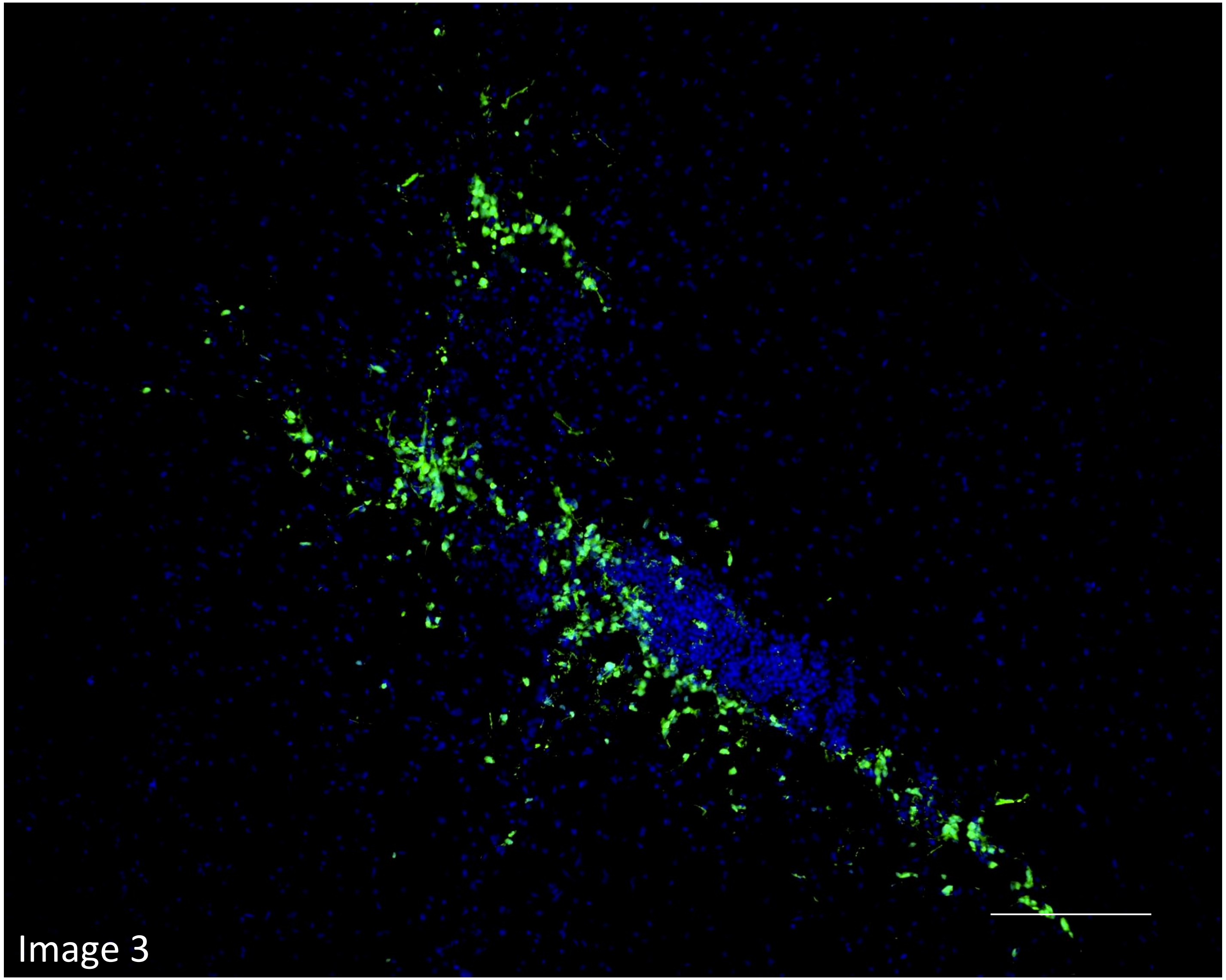

eGFP/TOPRO3 – day 5

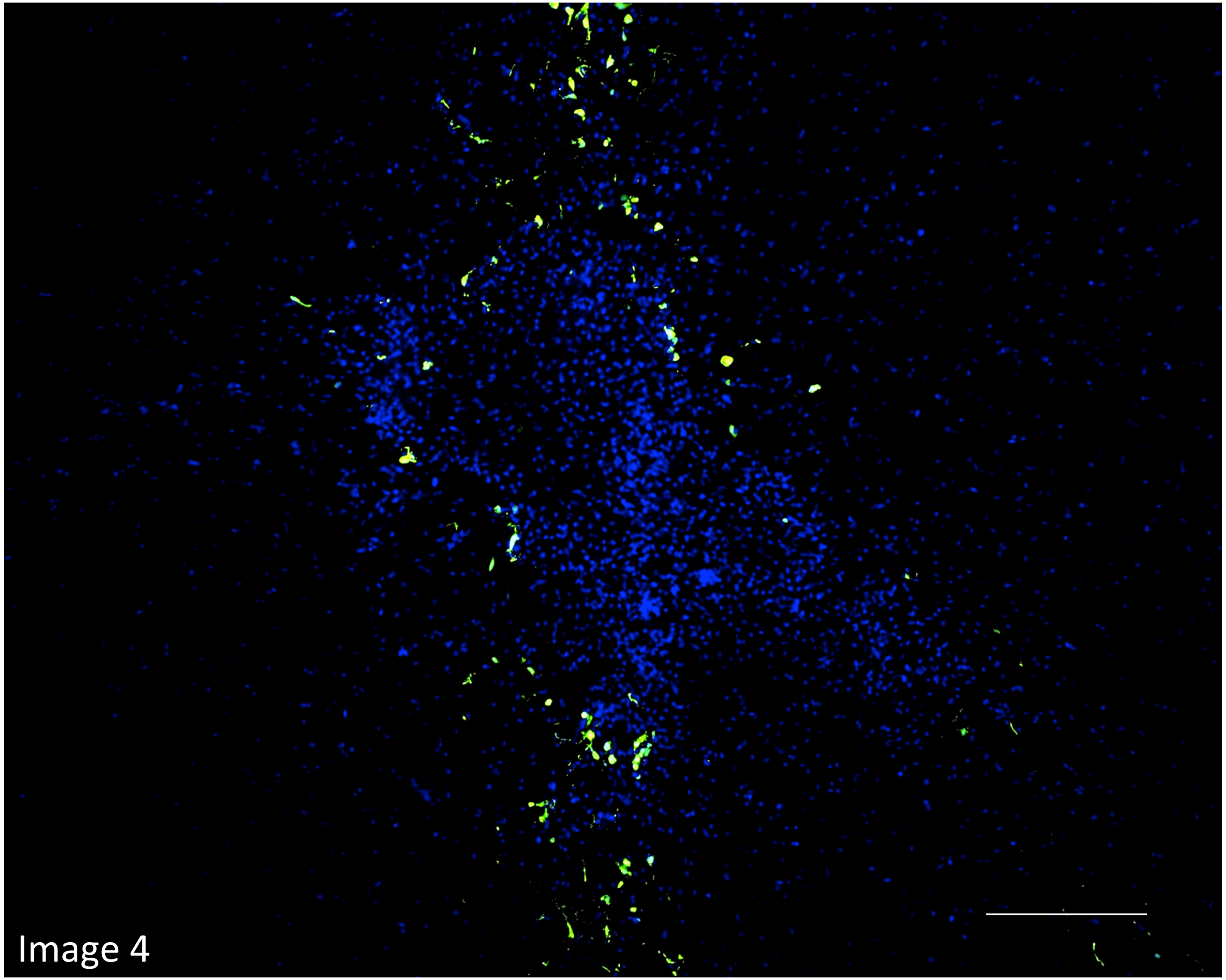

eGFP/TOPRO3 – day 7

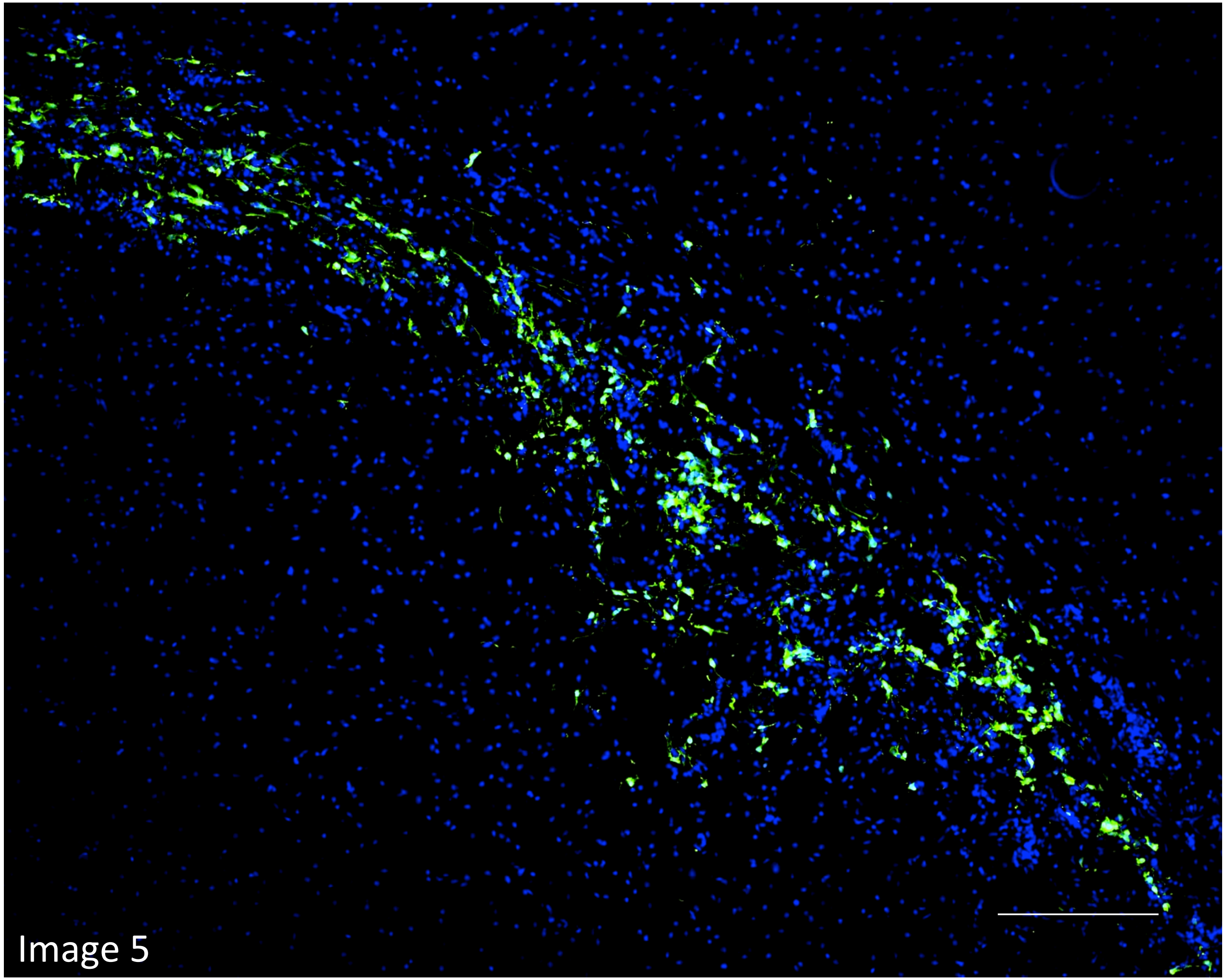

eGFP/TOPRO3 – day 14

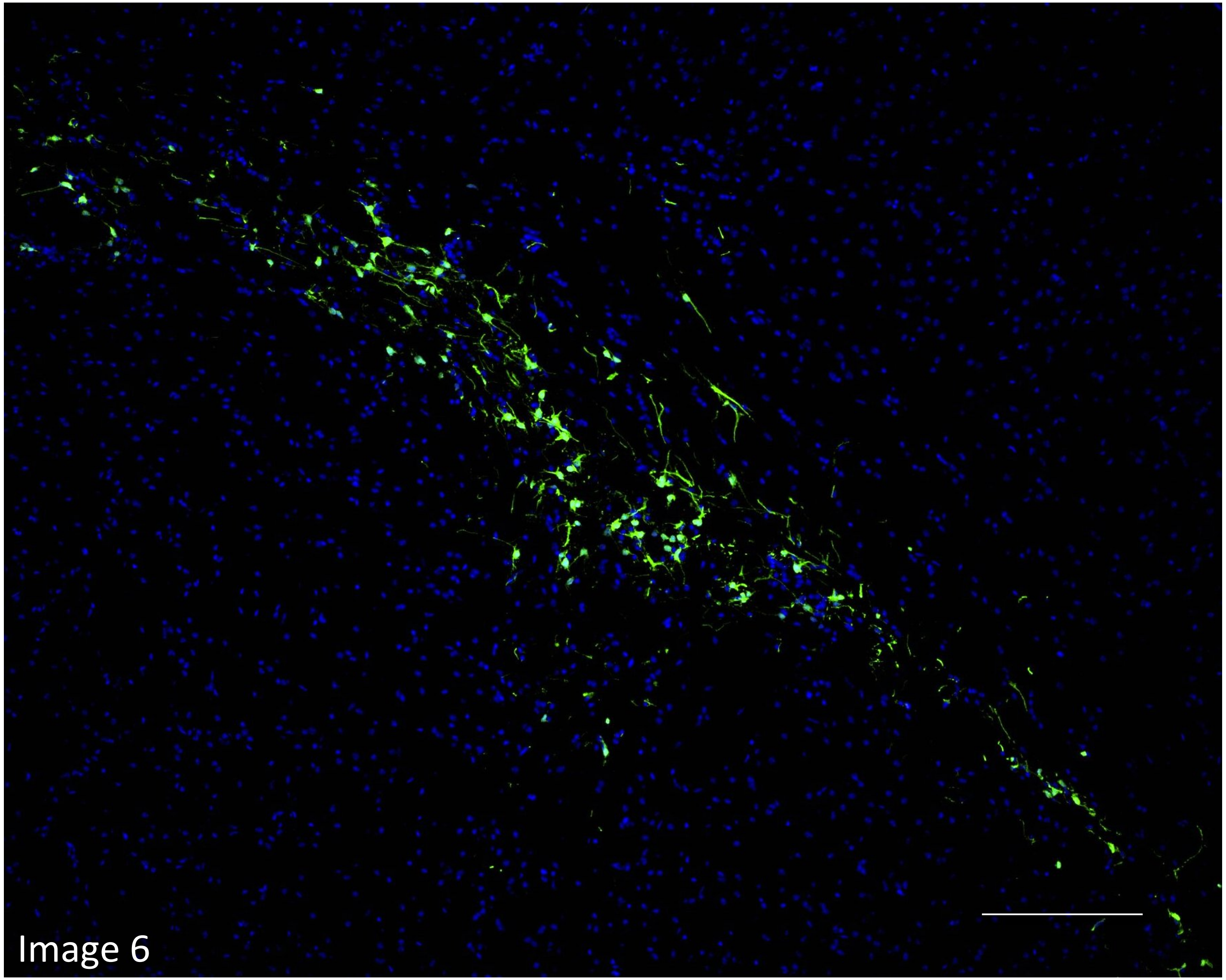

eGFP/Hypoxyprobe-1 – day 0

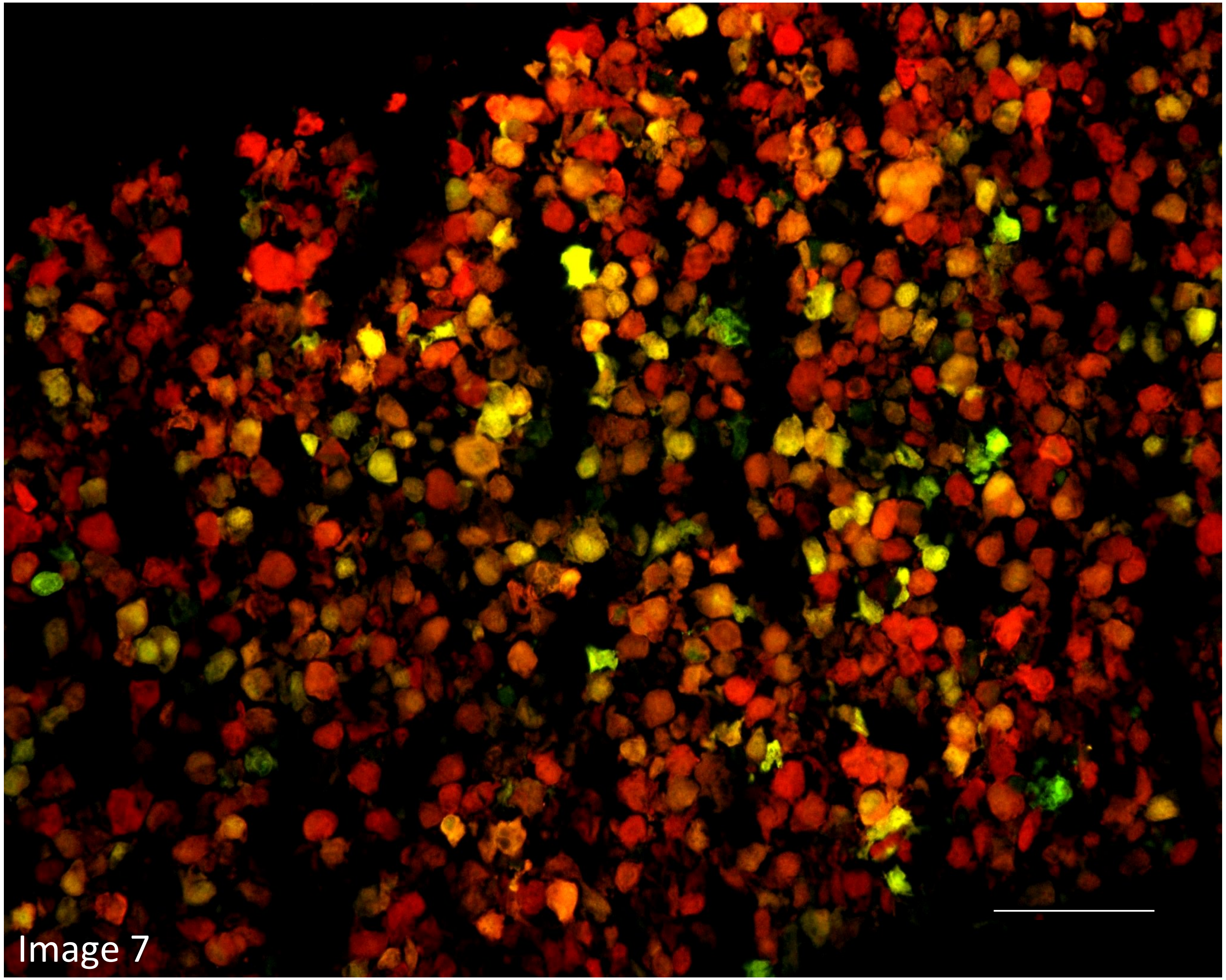

eGFP/Hypoxyprobe-1 – day 1

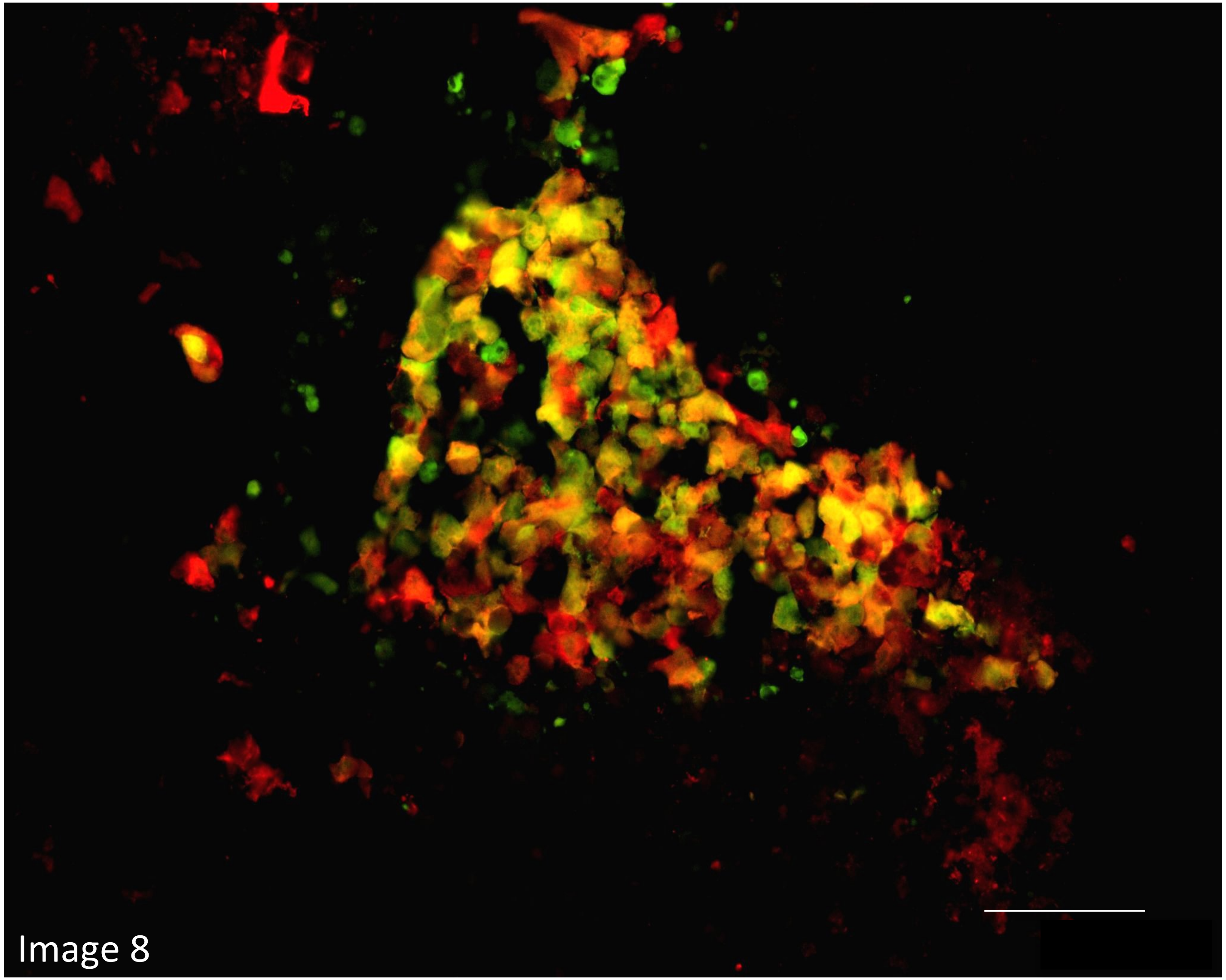

eGFP/Hypoxyprobe-1 – day 3

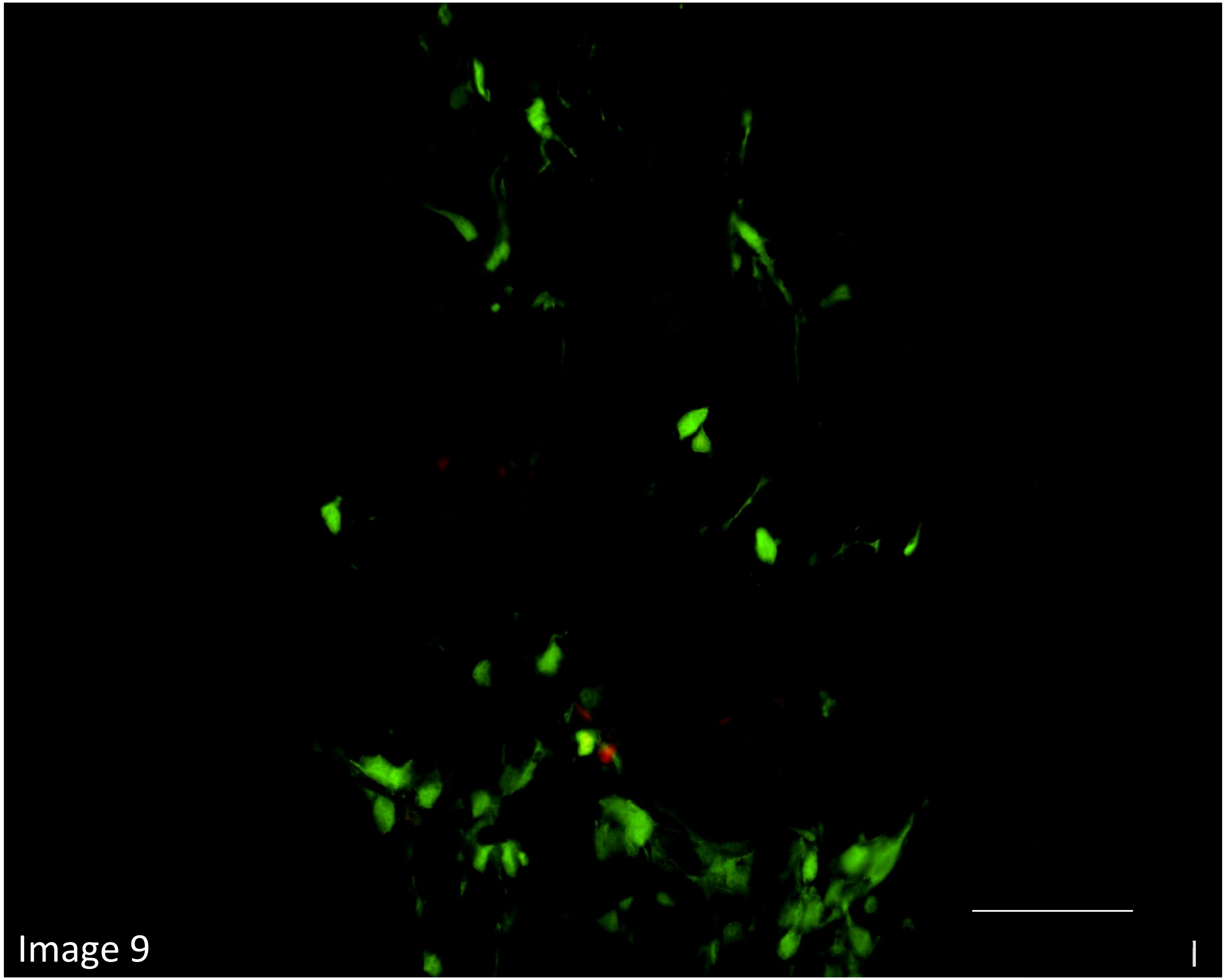

eGFP/Hypoxyprobe-1 – day 5

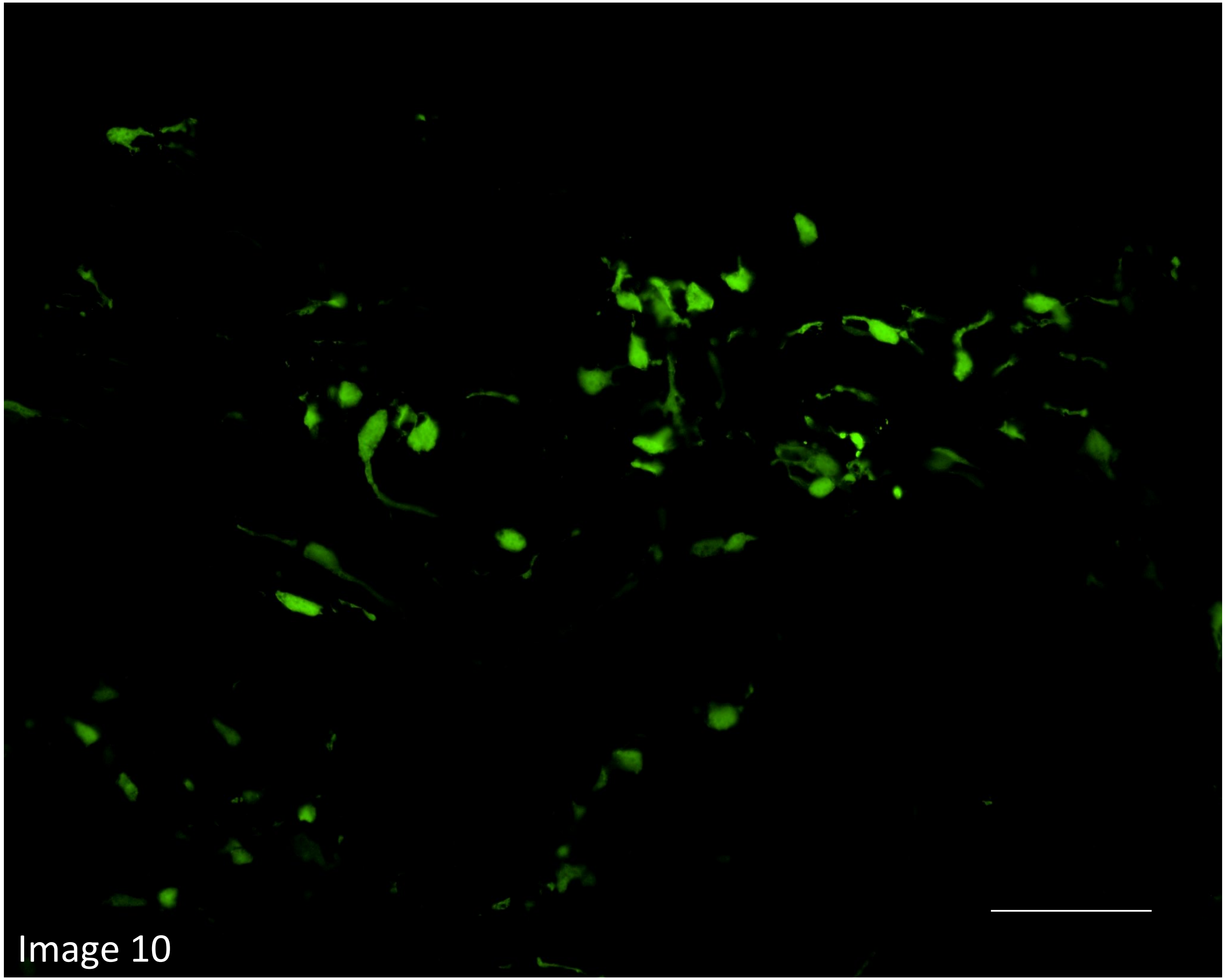

eGFP/Hypoxyprobe-1 – day 7

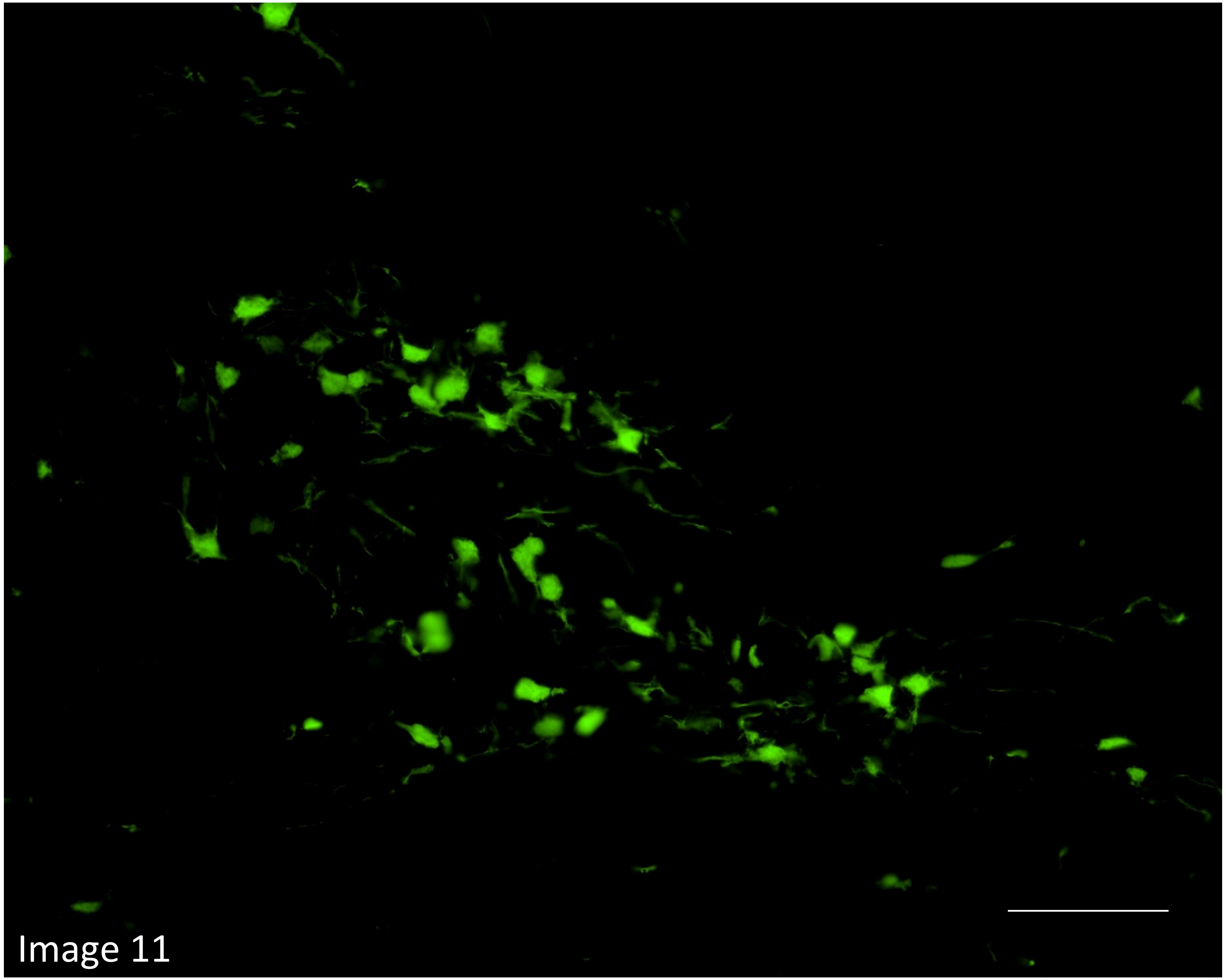

eGFP/Hypoxyprobe-1 – day 14

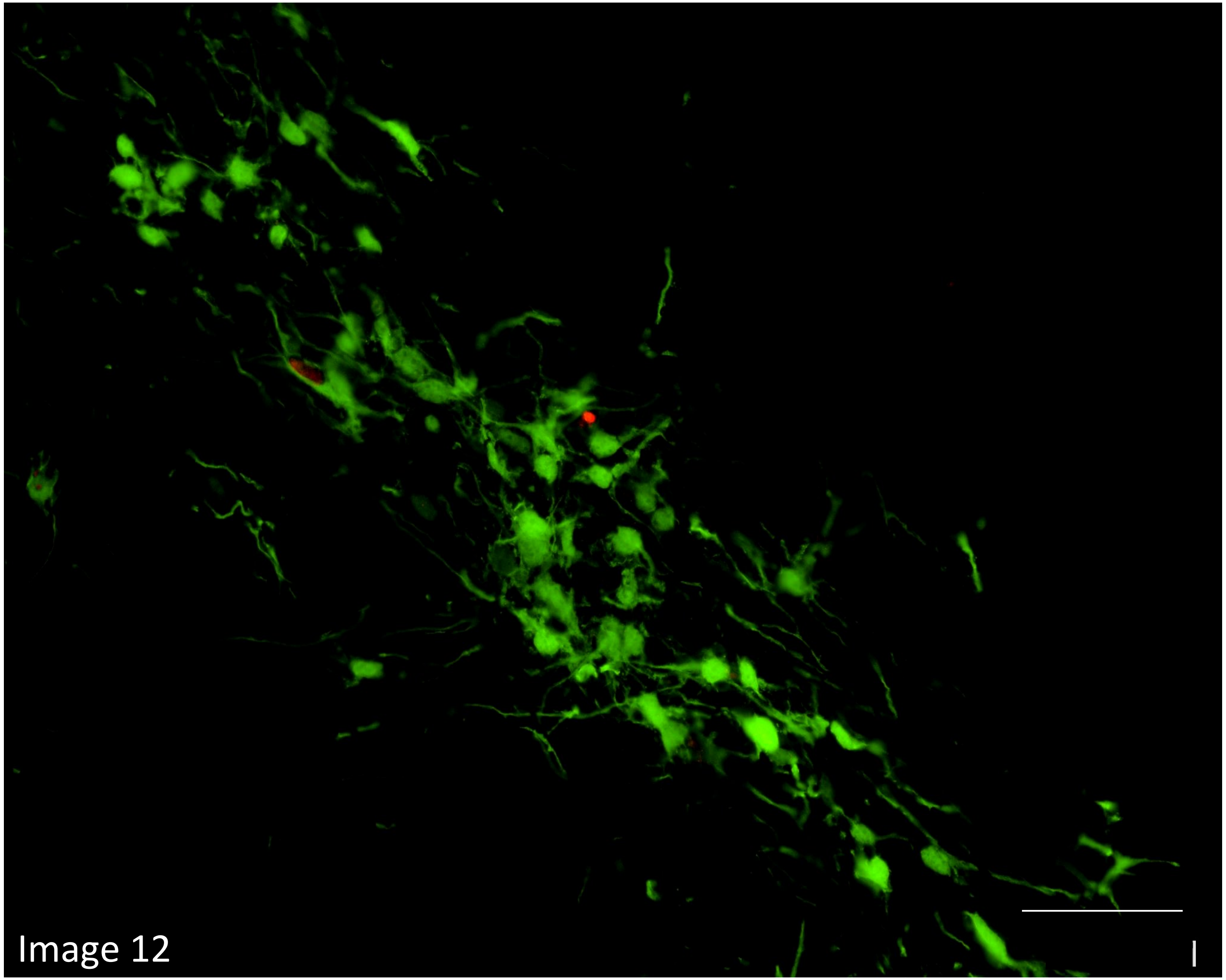

eGFP/MBP – day 0

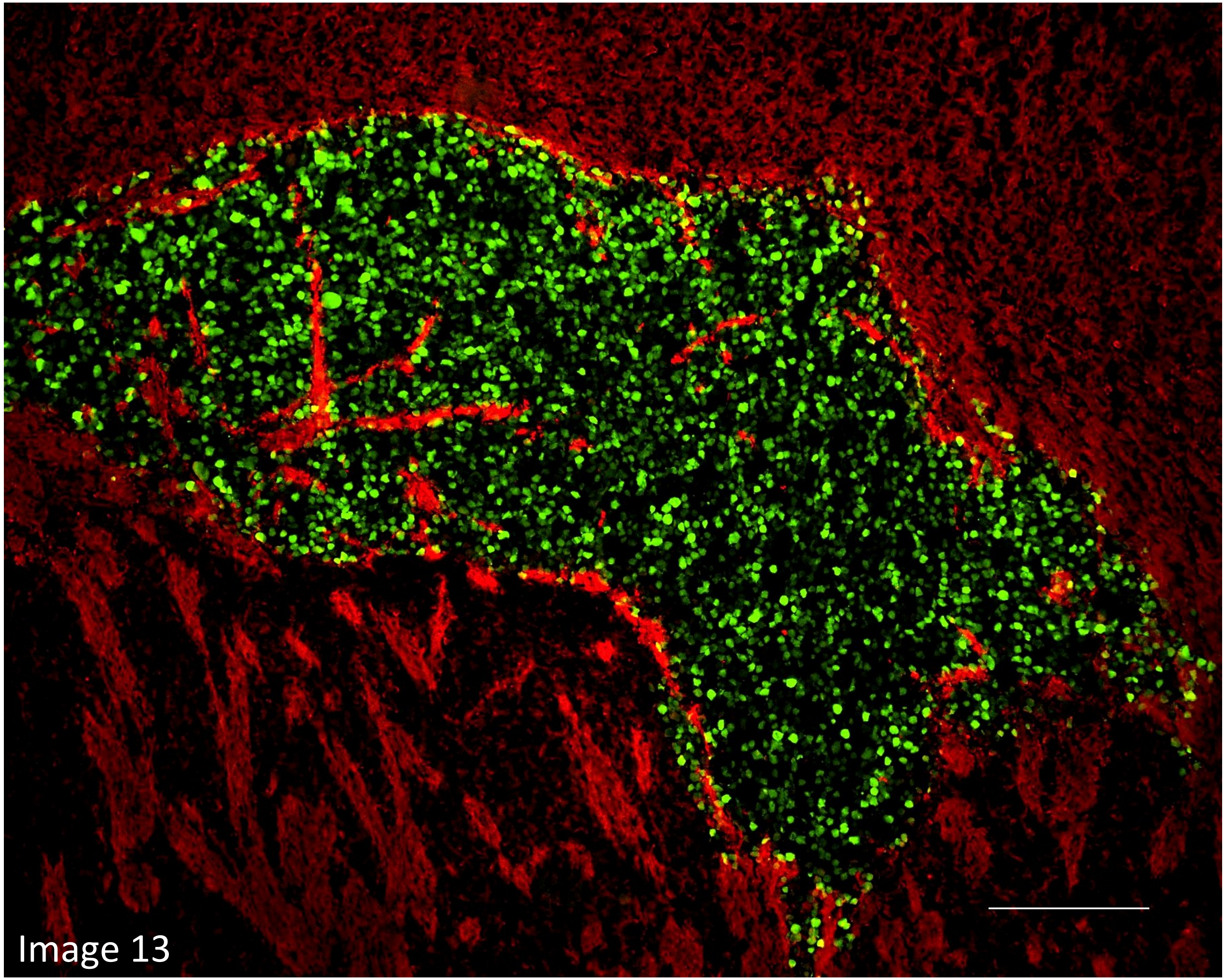

eGFP/MBP – day 1

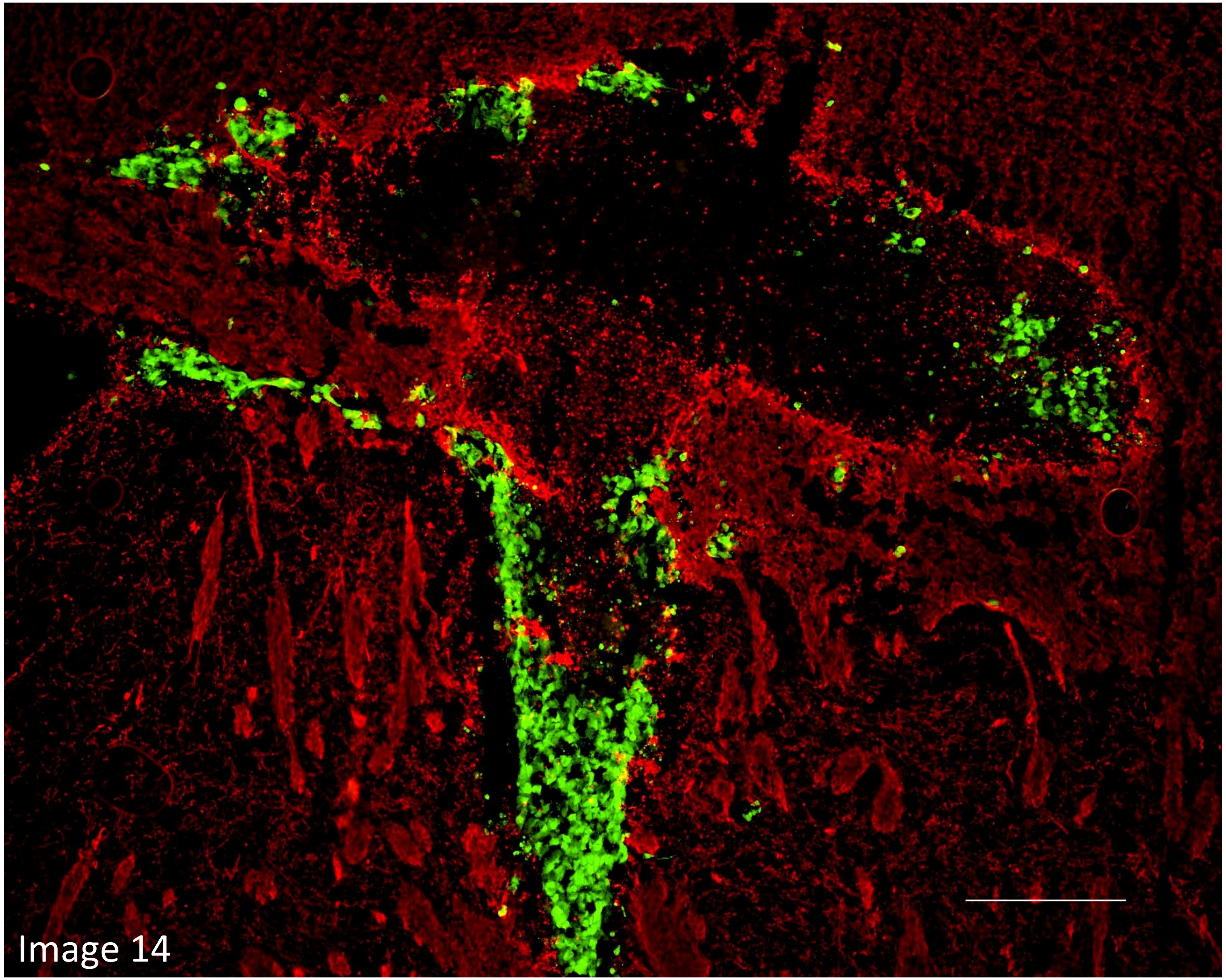

eGFP/MBP – day 3

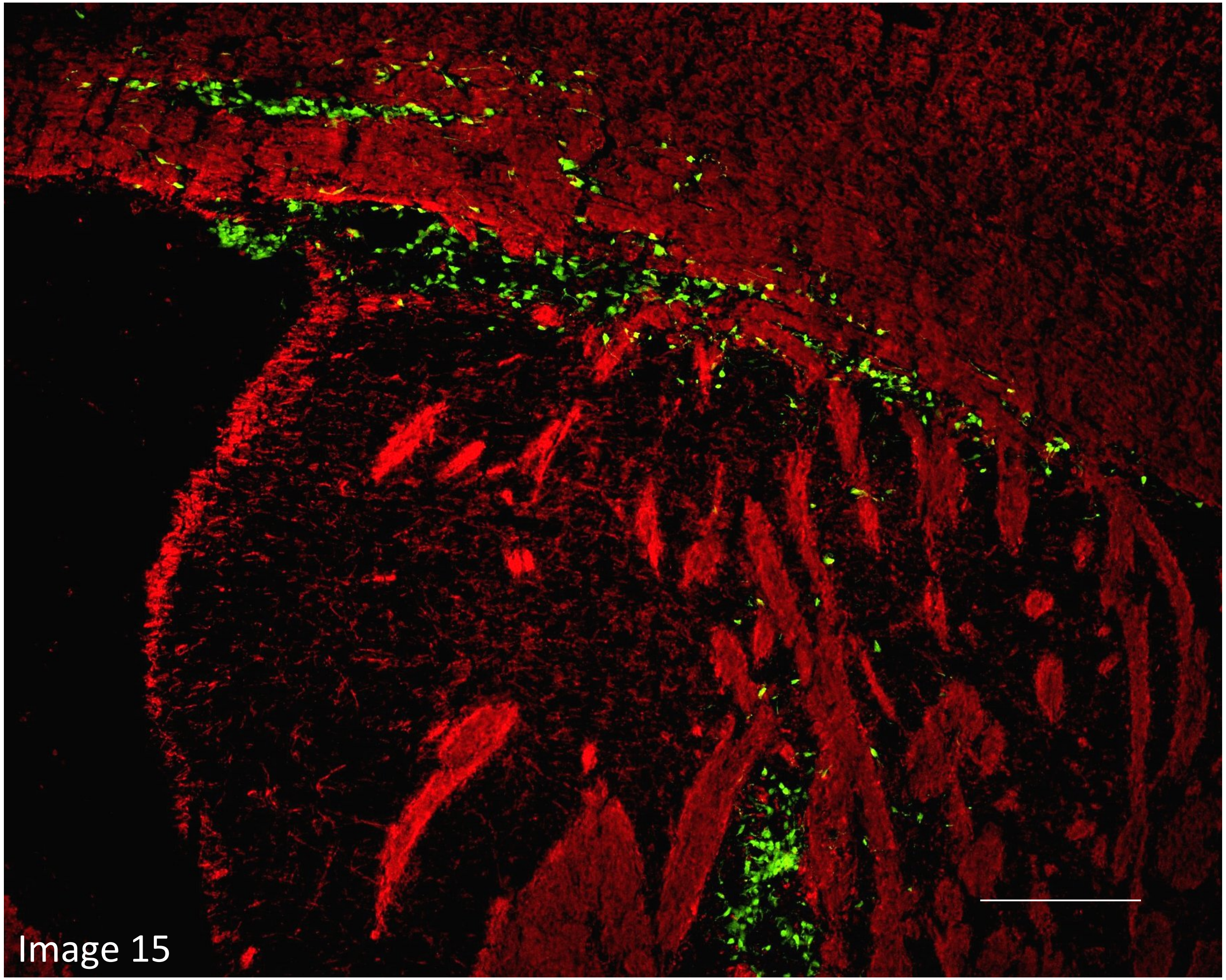

eGFP/MBP – day 5

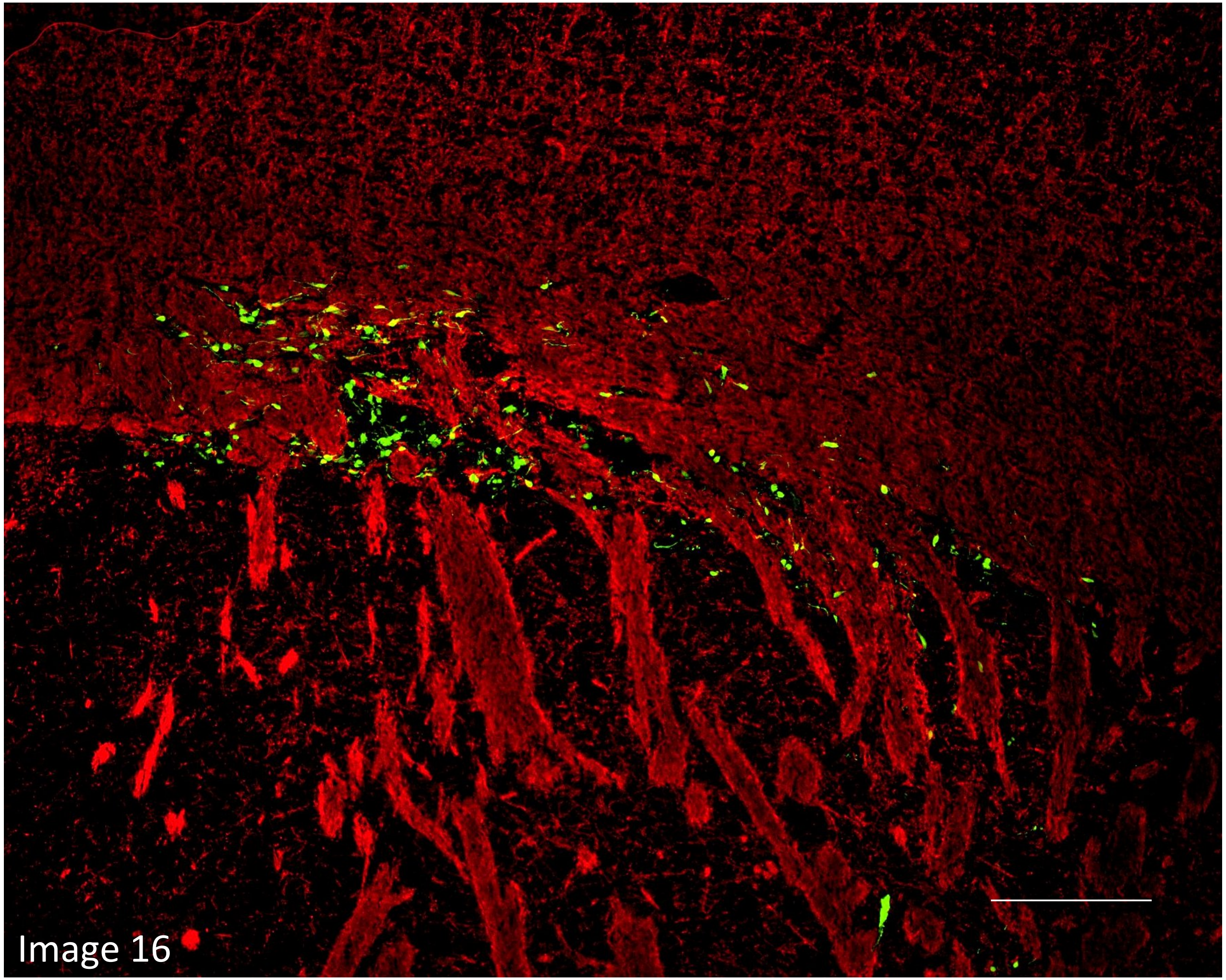

eGFP/MBP – day 7

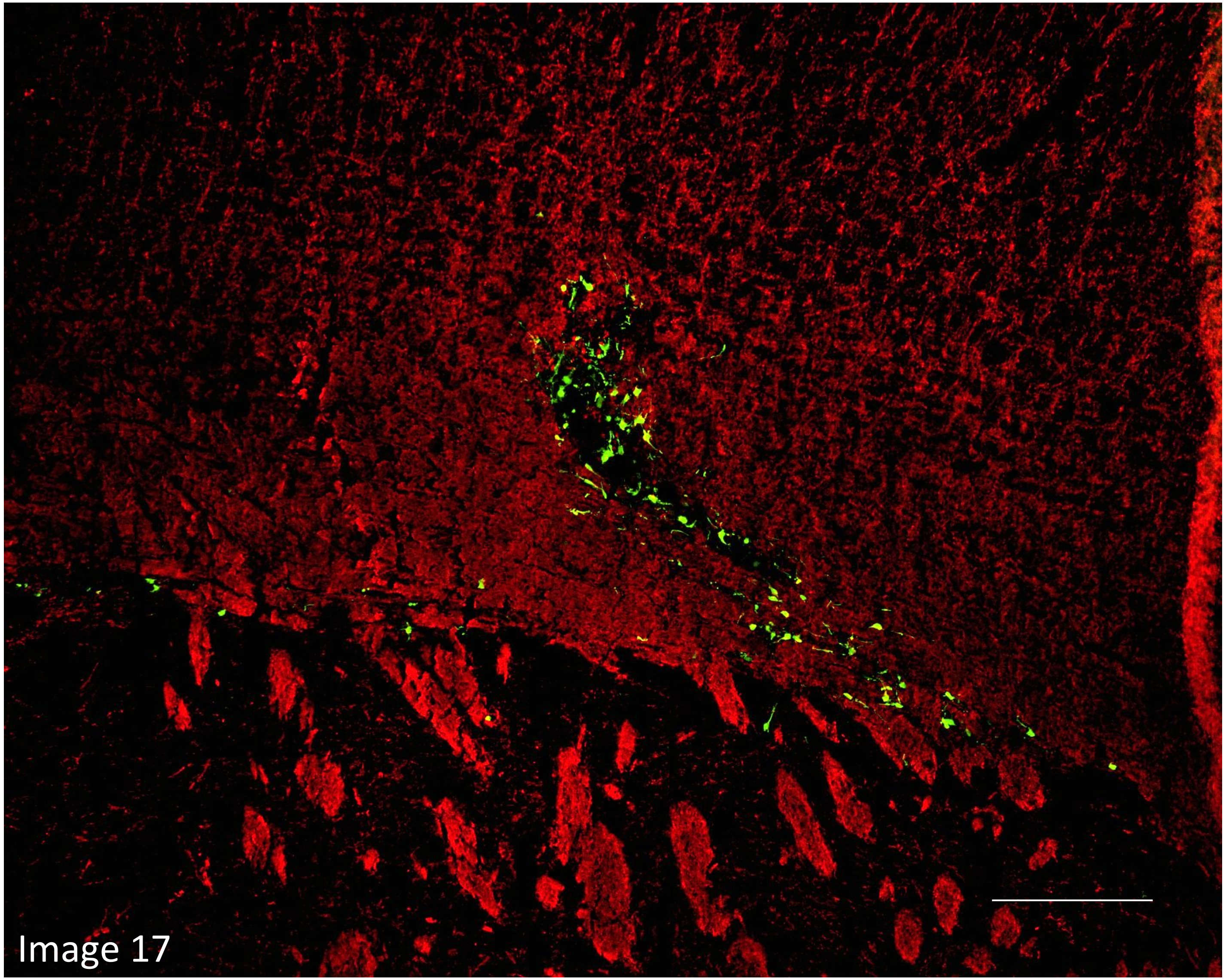

eGFP/MBP – day 14

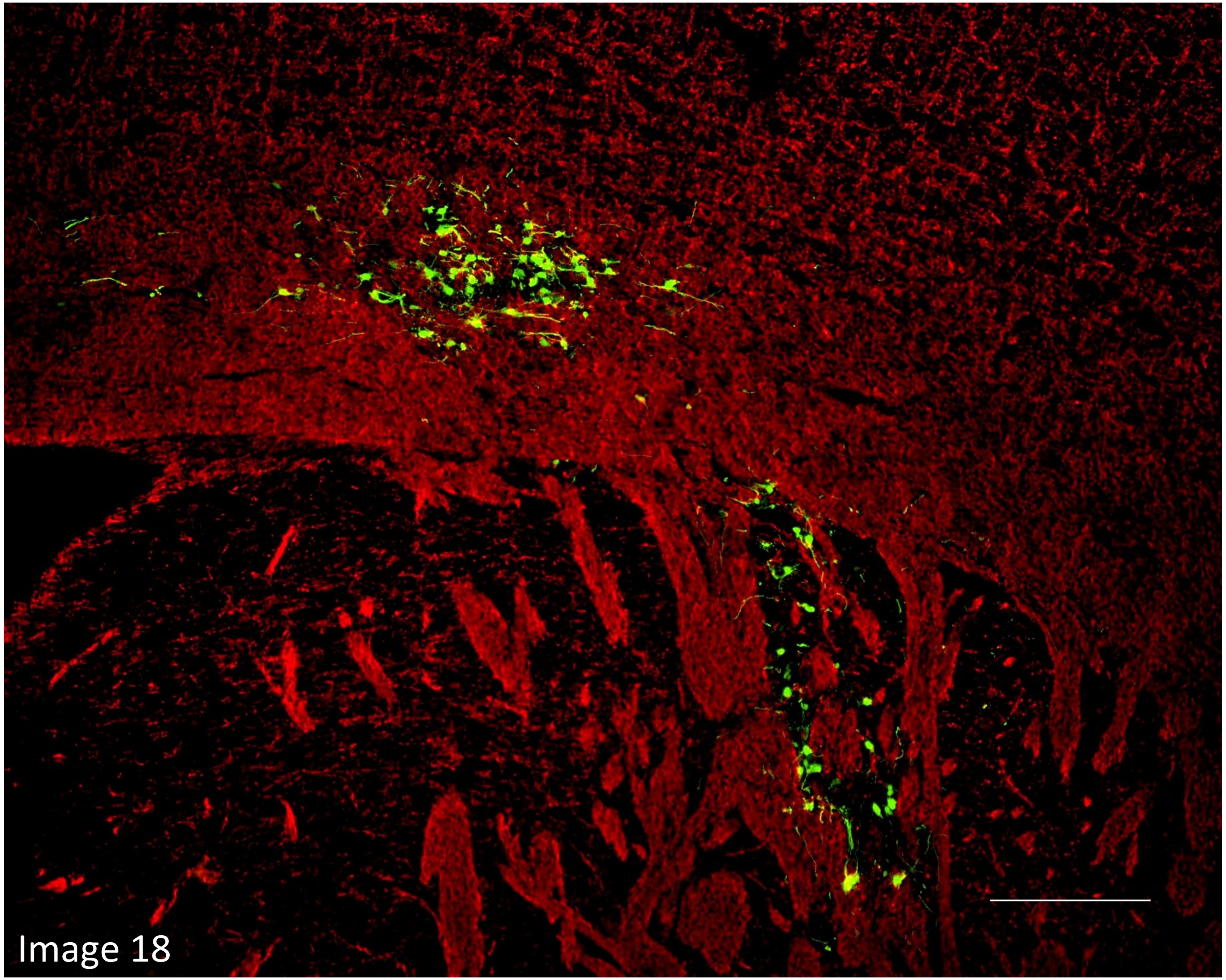

eGFP/TOPRO3/Iba-1 – day 0

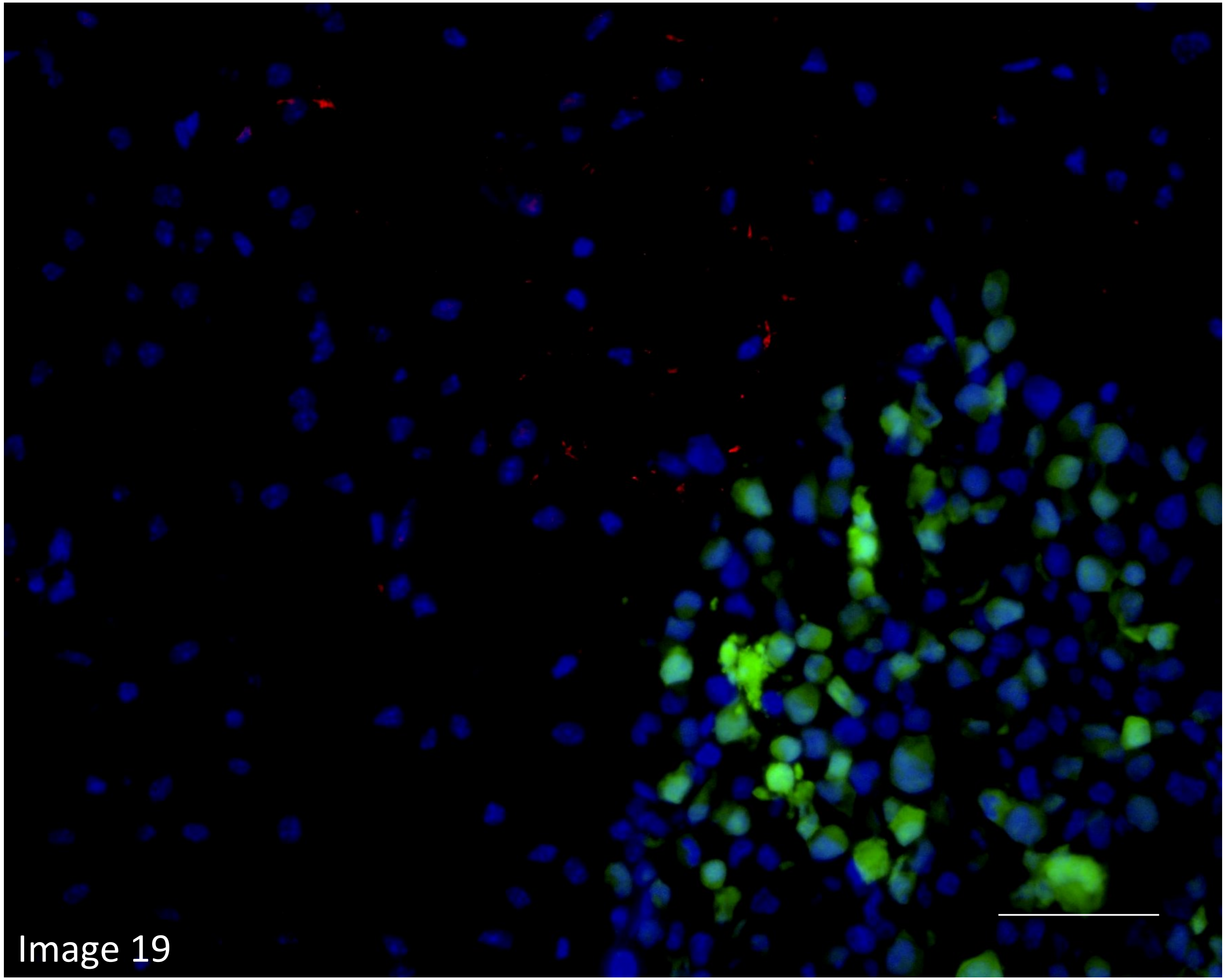

eGFP/TOPRO3/Iba-1 – day 1

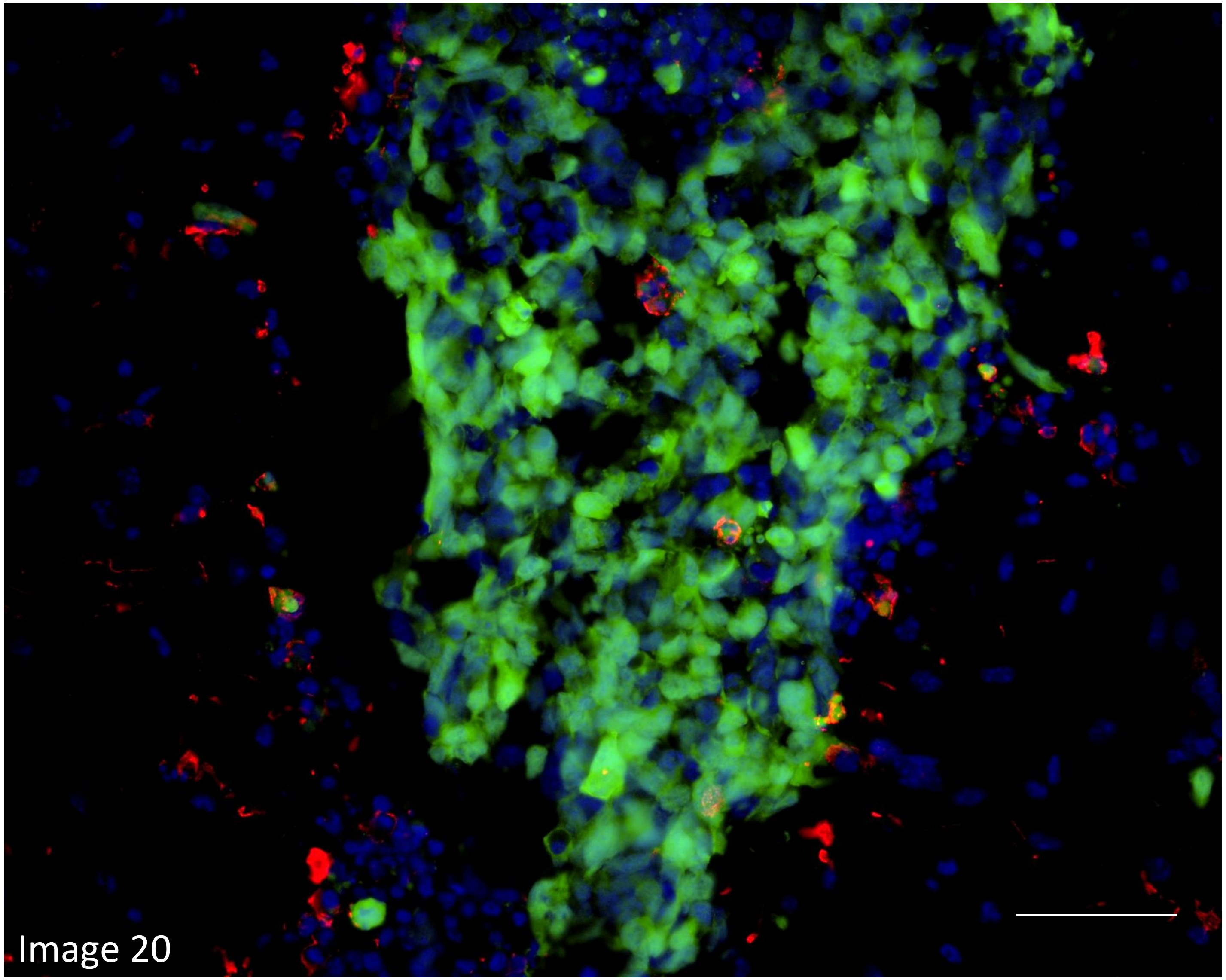

eGFP/TOPRO3/Iba-1 – day 3

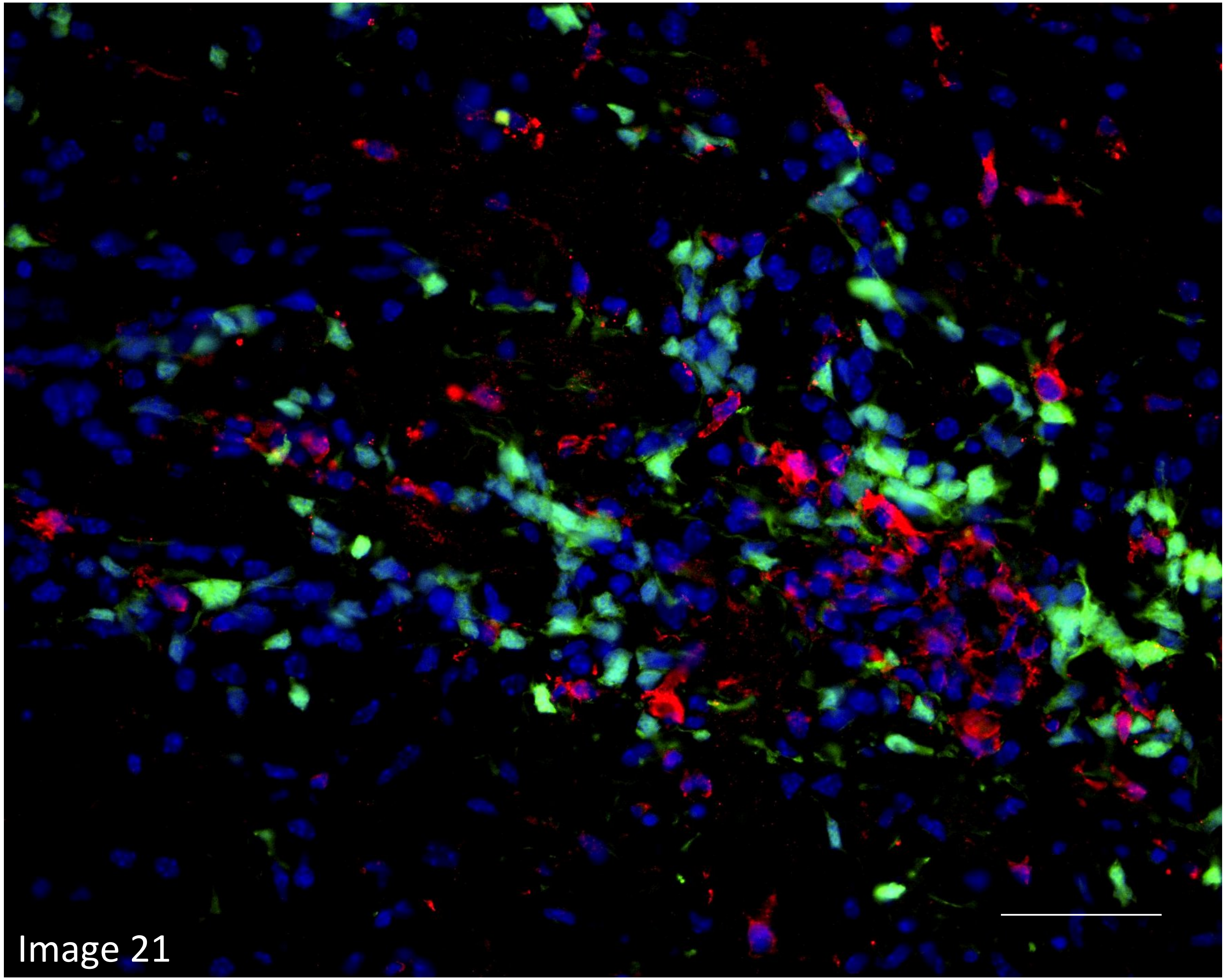

eGFP/TOPRO3/Iba-1 – day 5

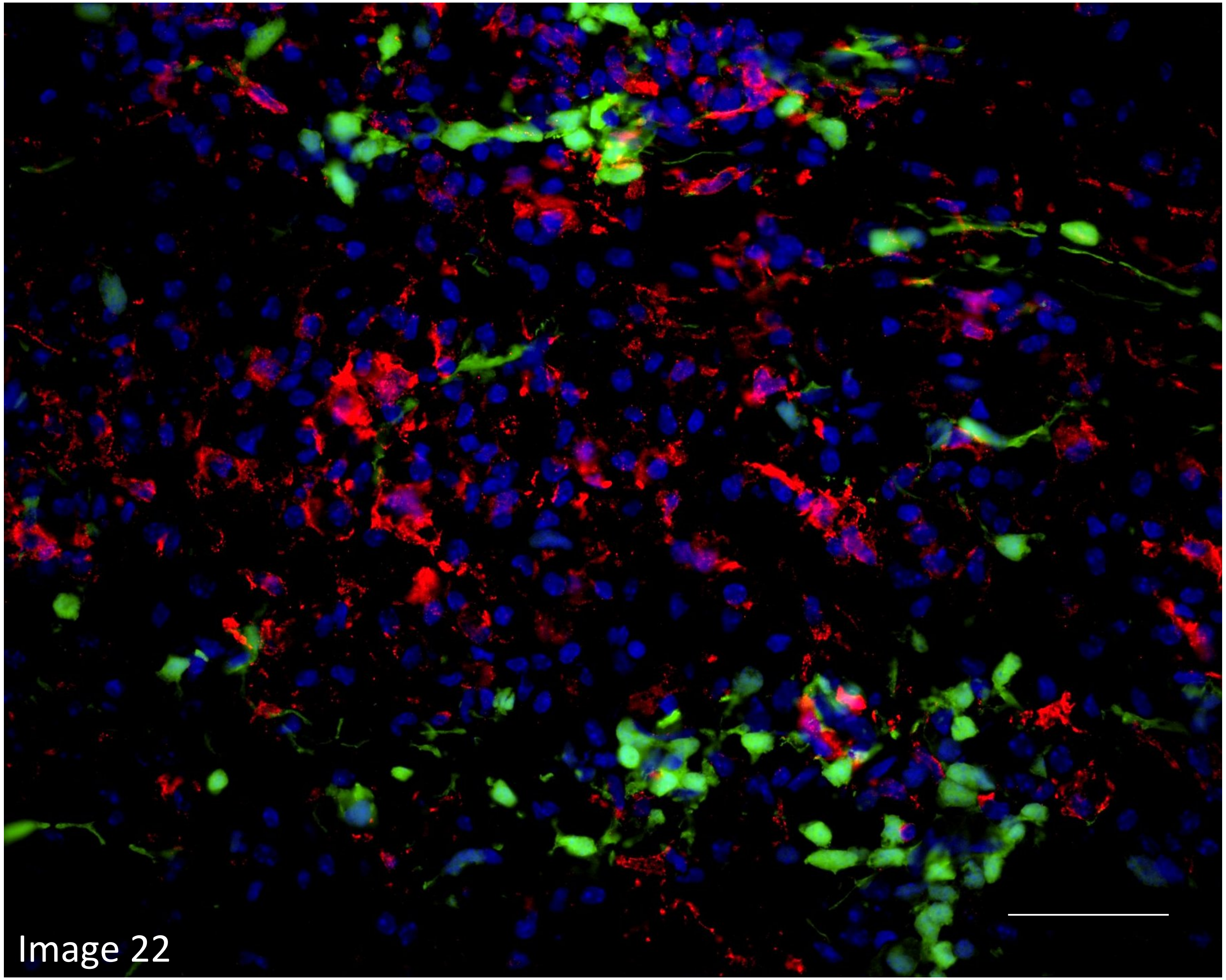

eGFP/TOPRO3/Iba-1 – day 7

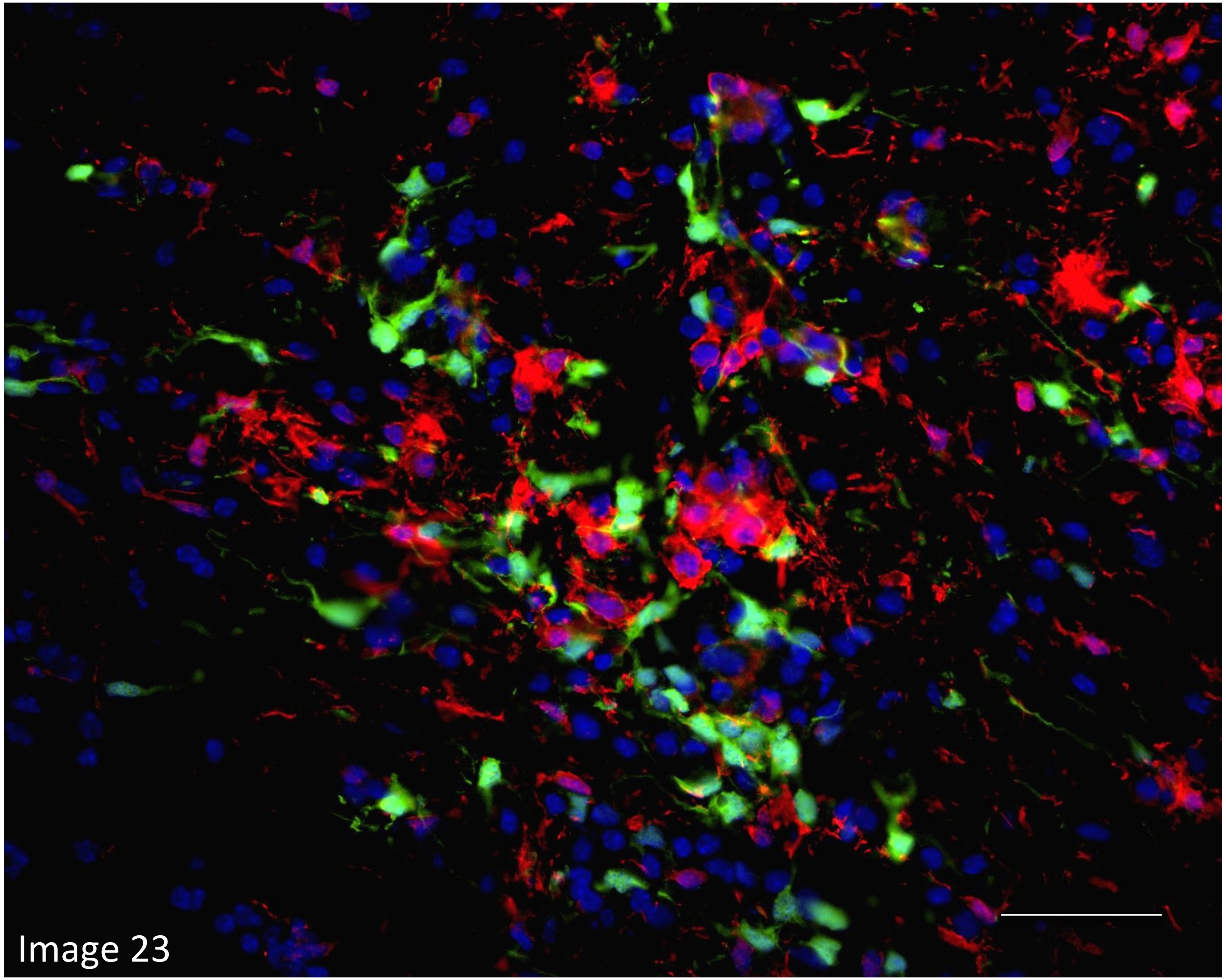

eGFP/TOPRO3/Iba-1 – day 14

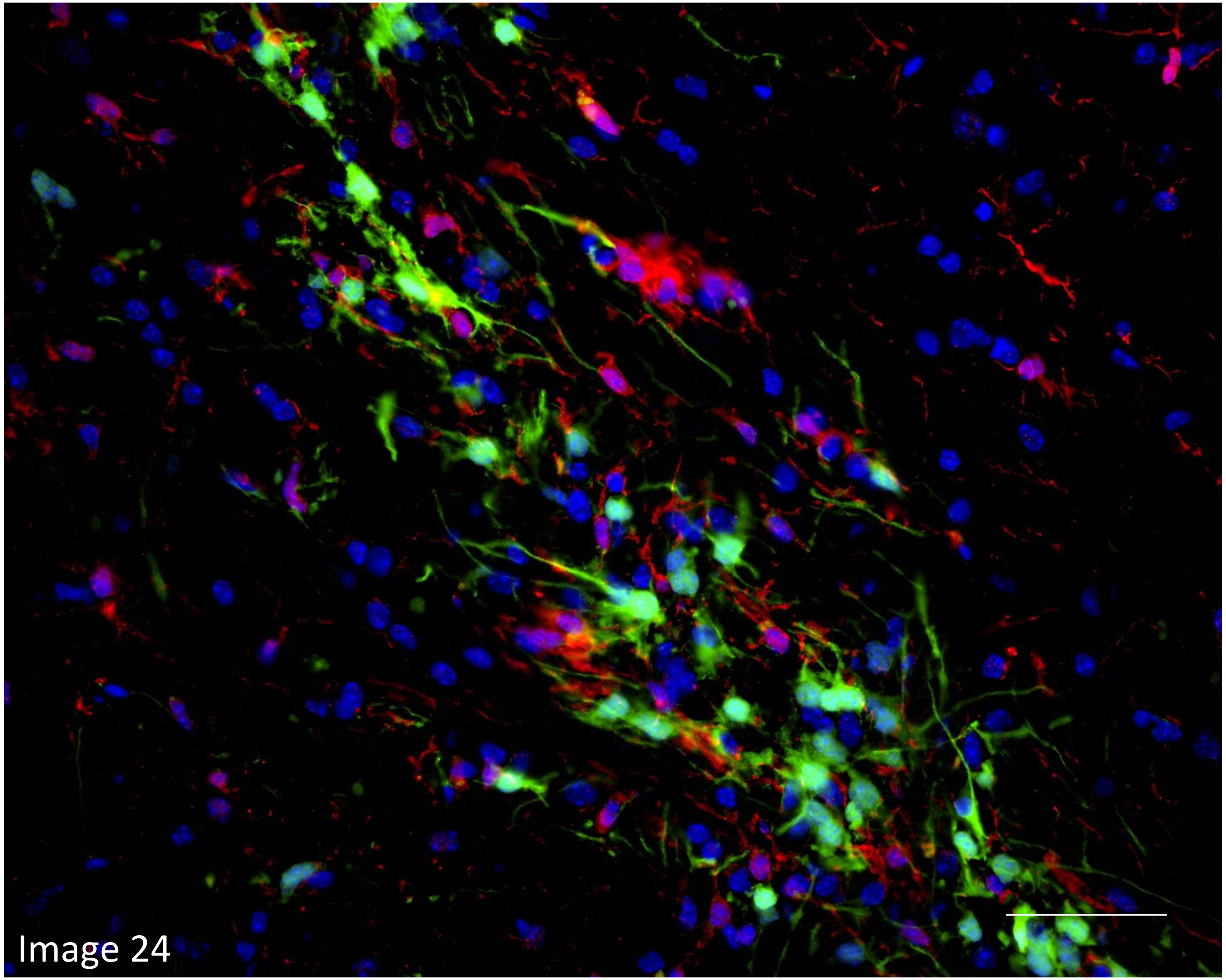

eGFP/TOPRO3/S100B – day 0

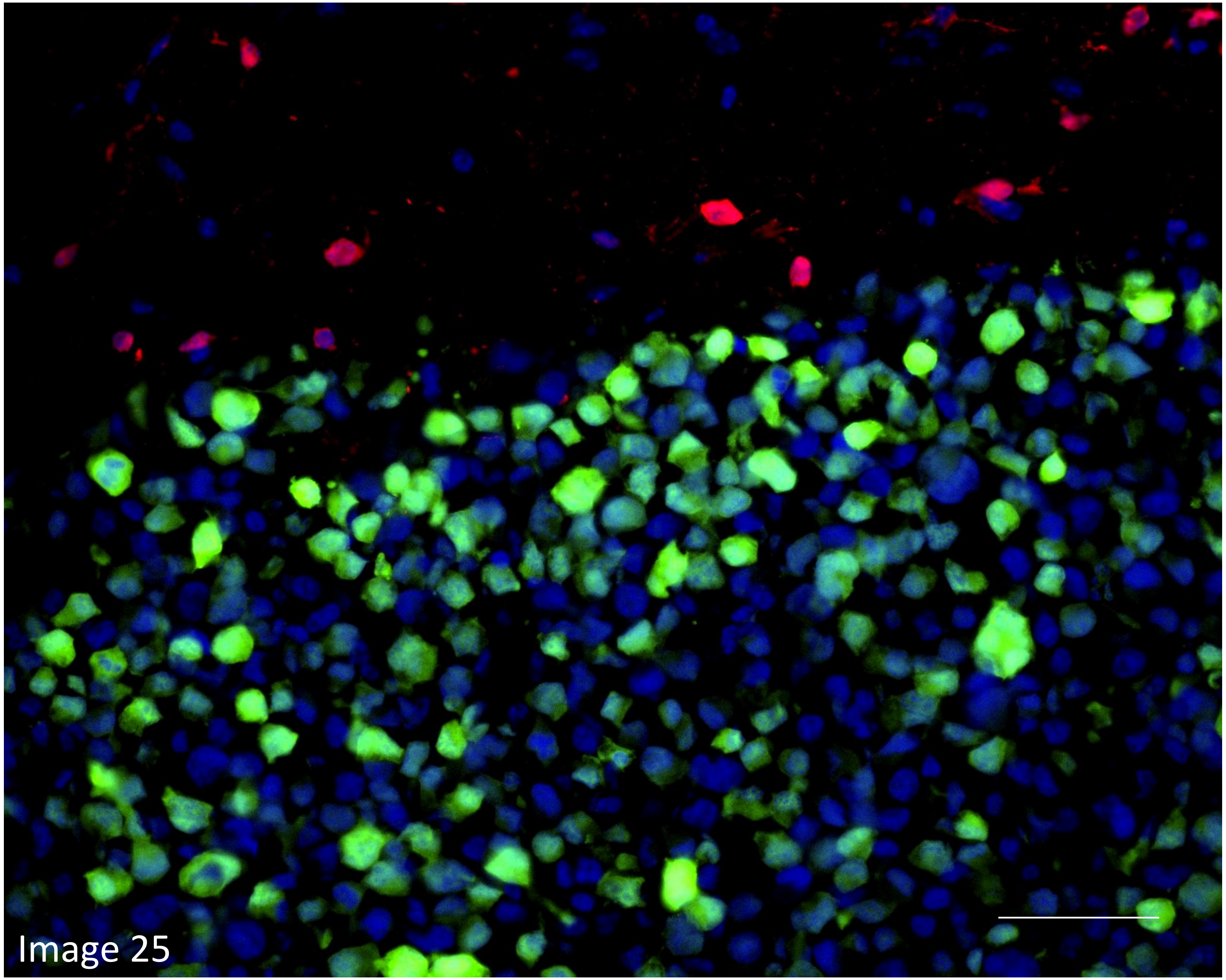

eGFP/TOPRO3/S100B – day 1

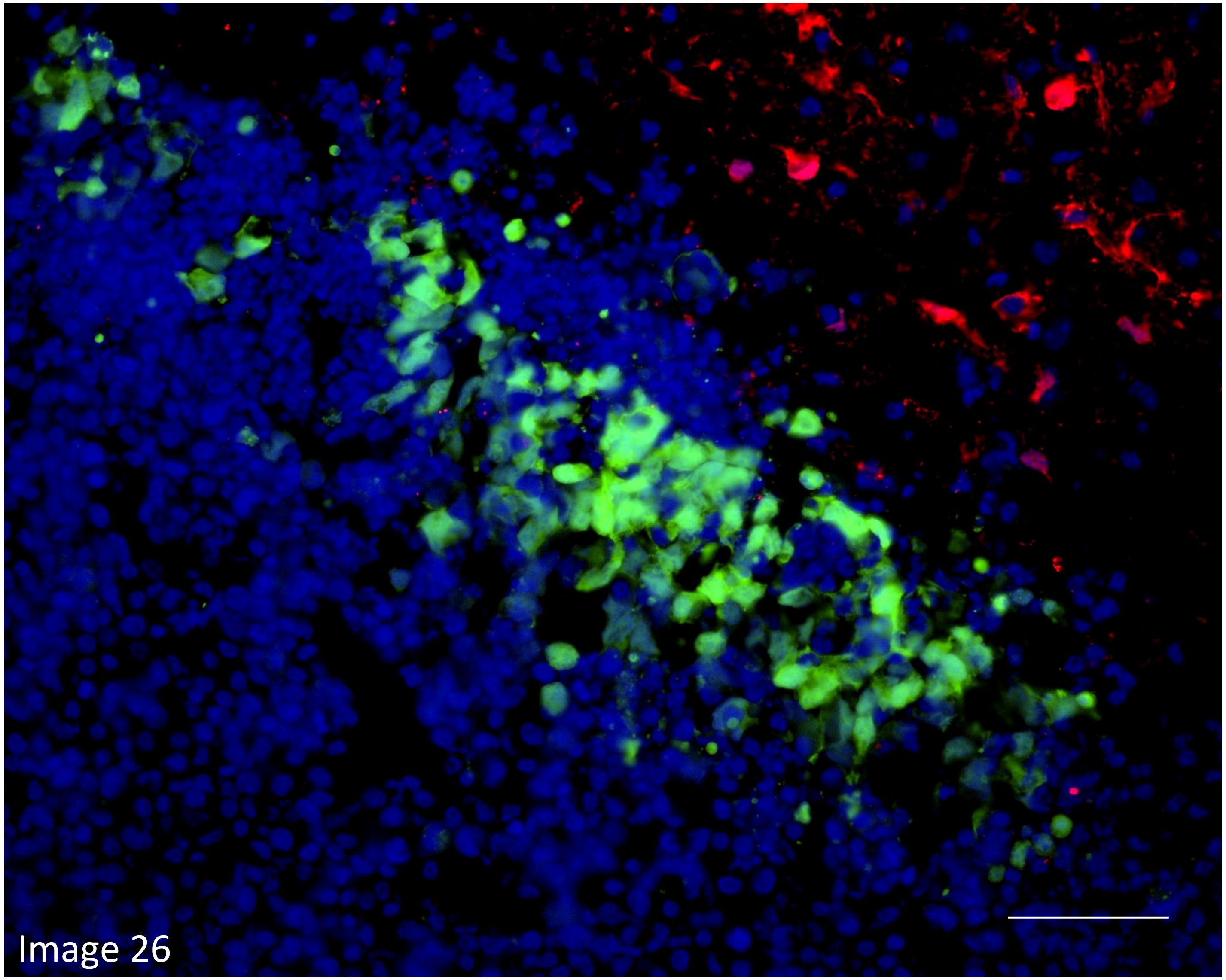

eGFP/TOPRO3/S100B – day 3

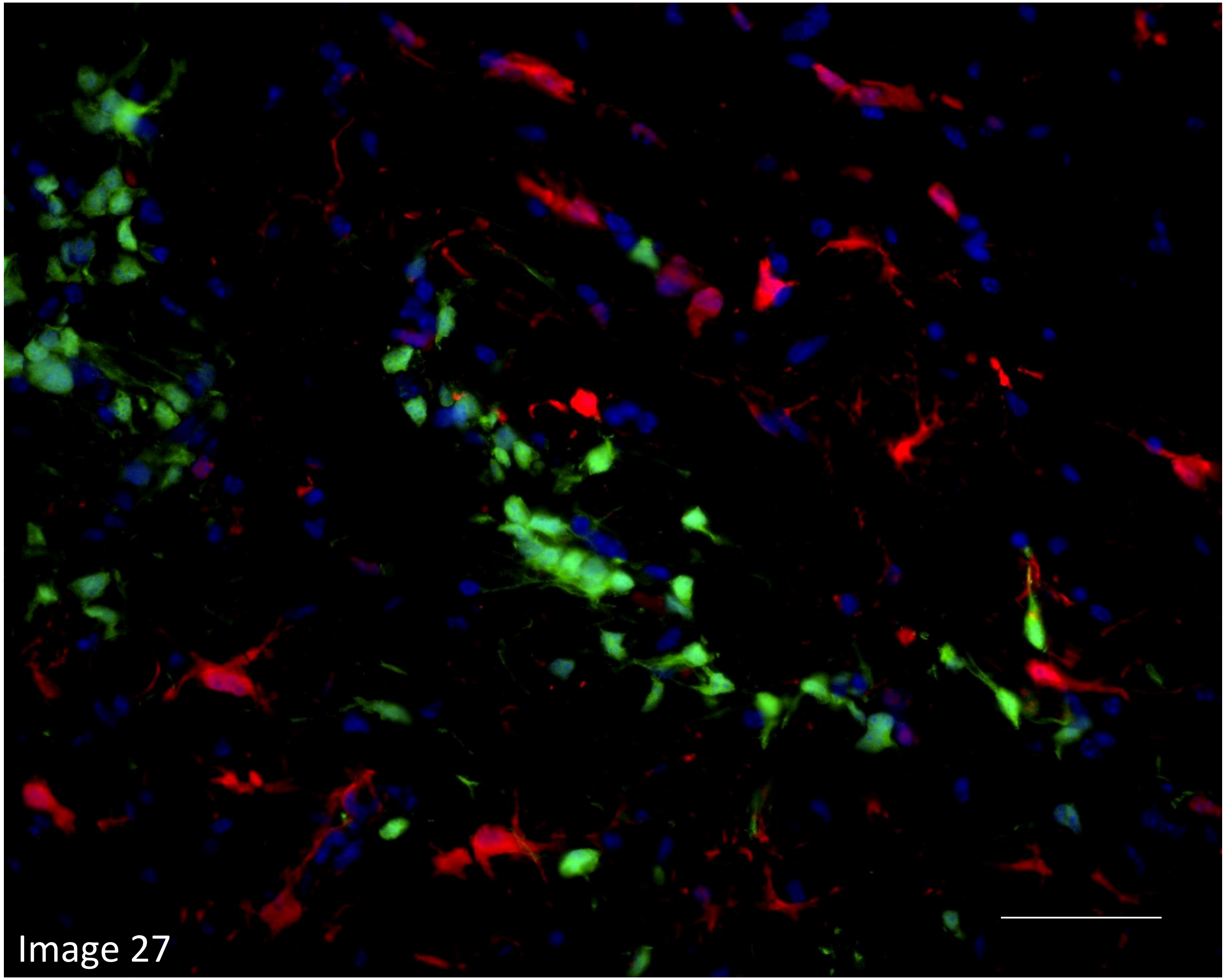

eGFP/TOPRO3/S100B – day 5

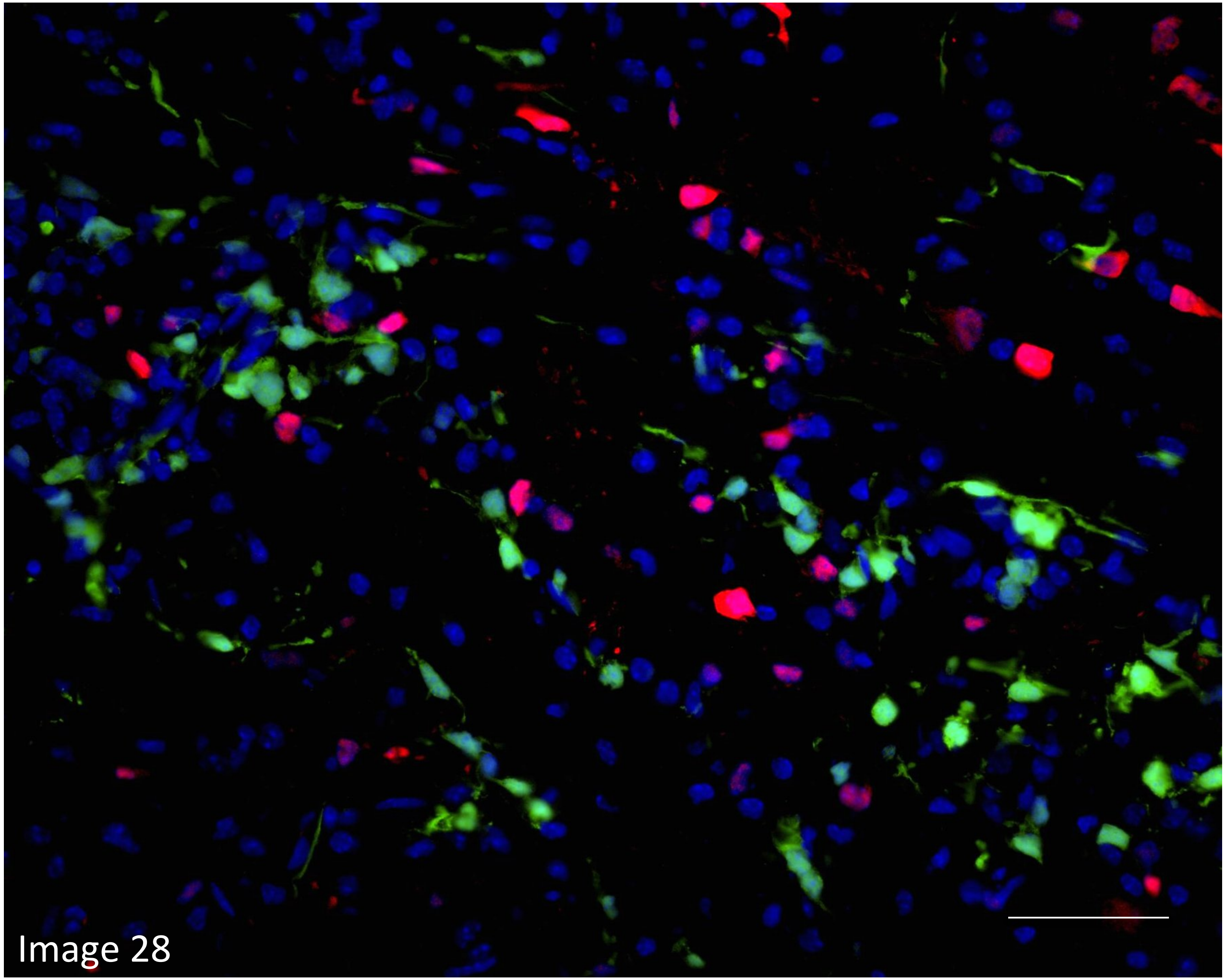

eGFP/TOPRO3/S100B – day 7

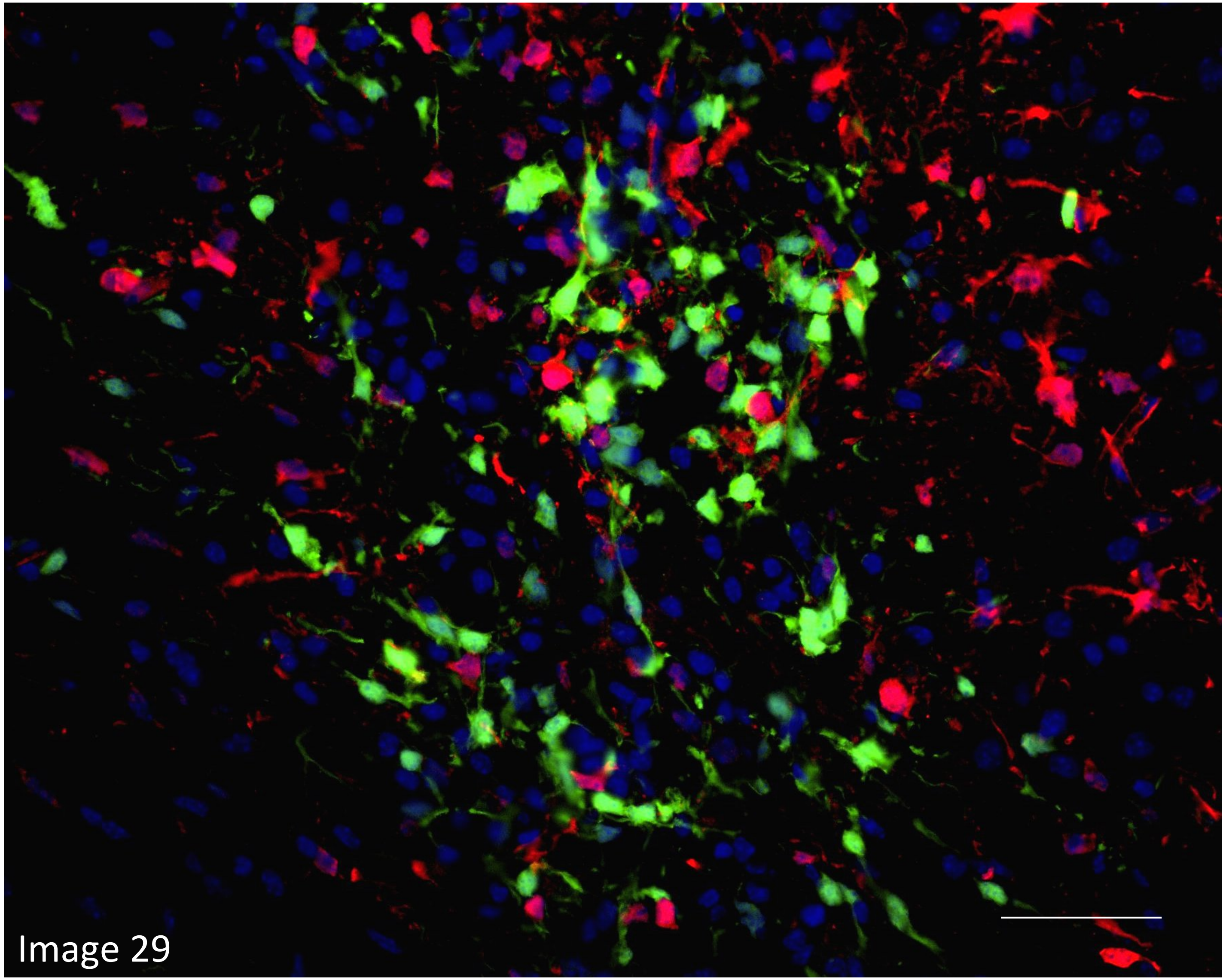

eGFP/TOPRO3/S100B – day 14

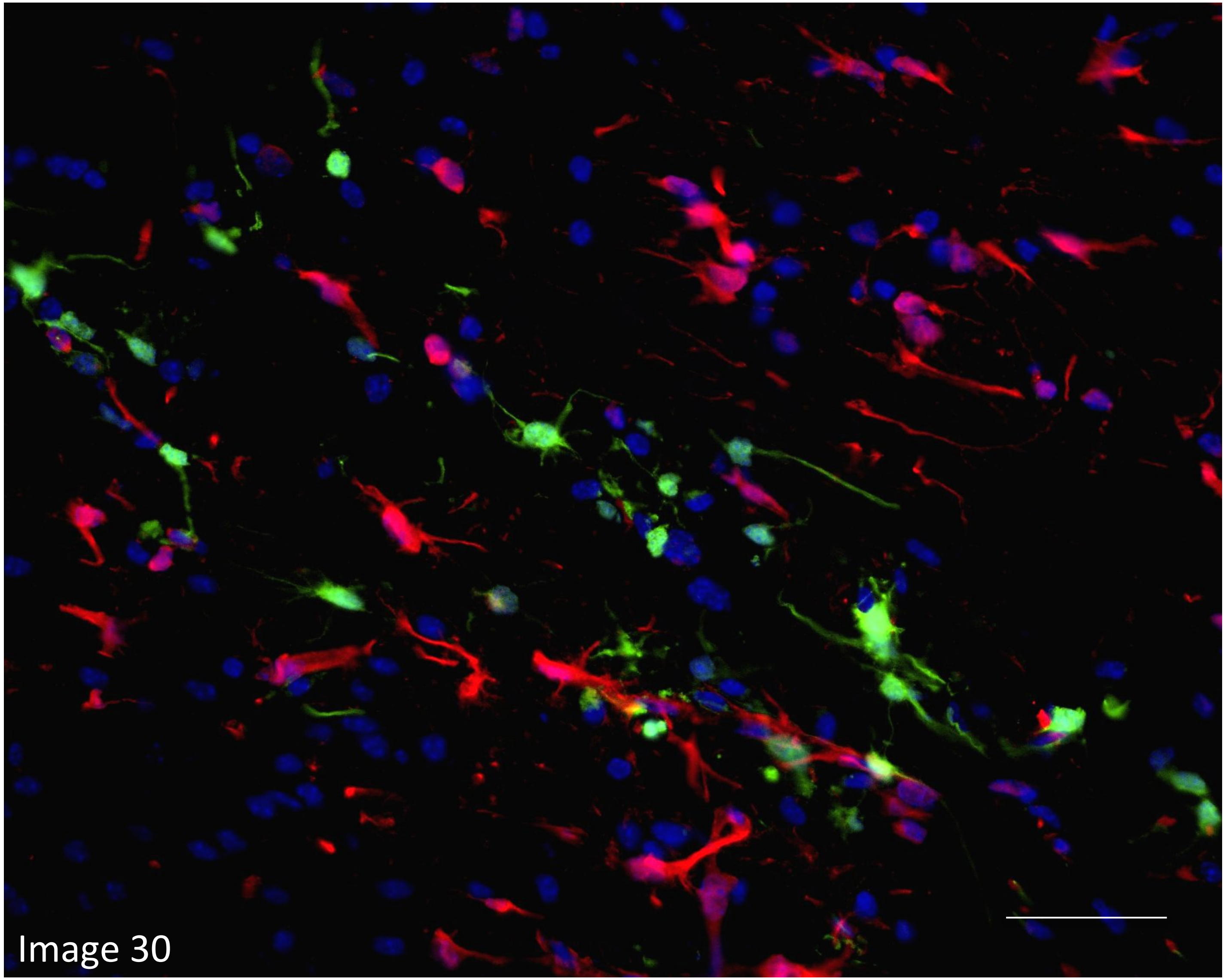

eGFP/TOPRO3/GFAP – day 0

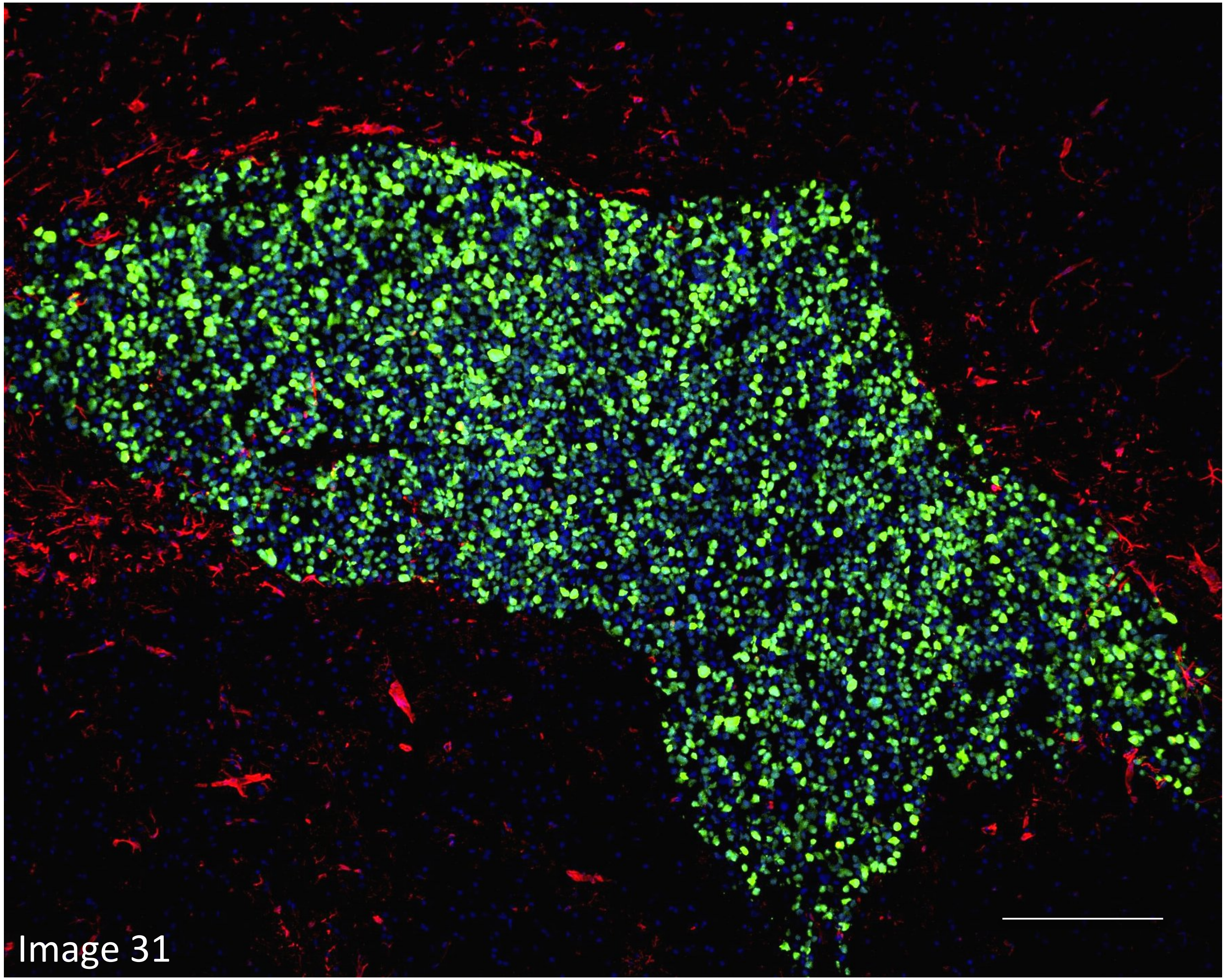

eGFP/TOPRO3/GFAP – day 1

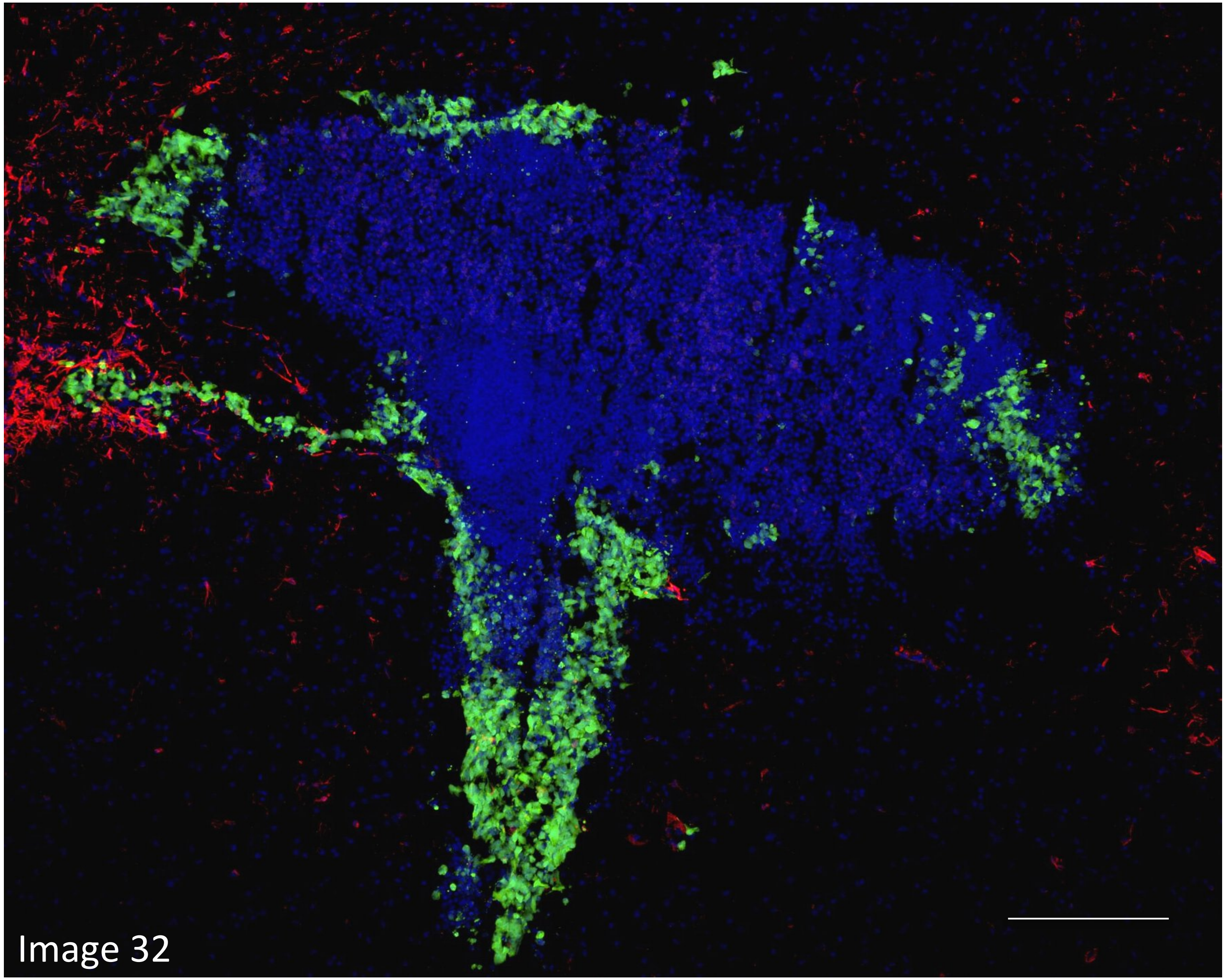

eGFP/TOPRO3/GFAP – day 3

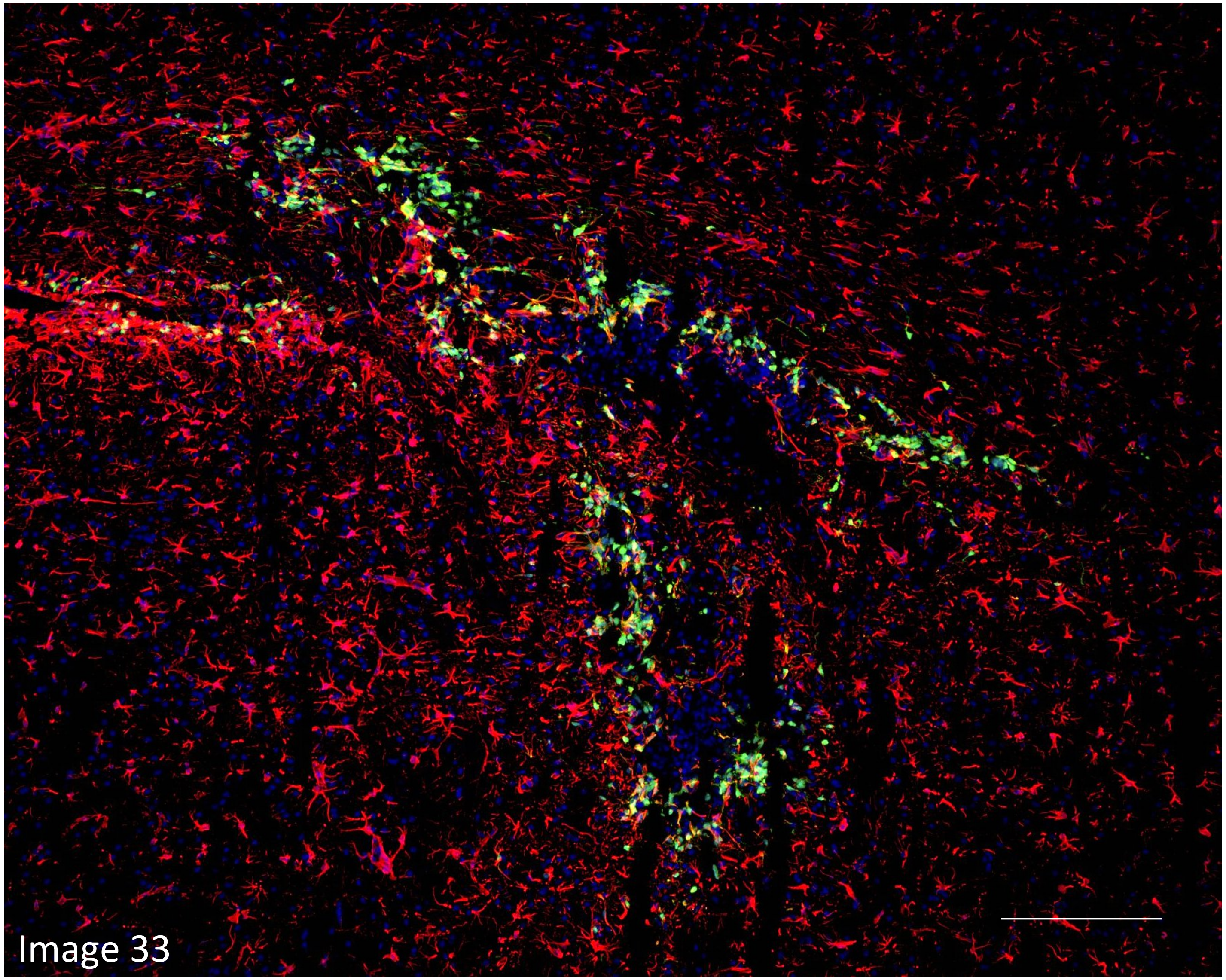

eGFP/TOPRO3/GFAP – day 5

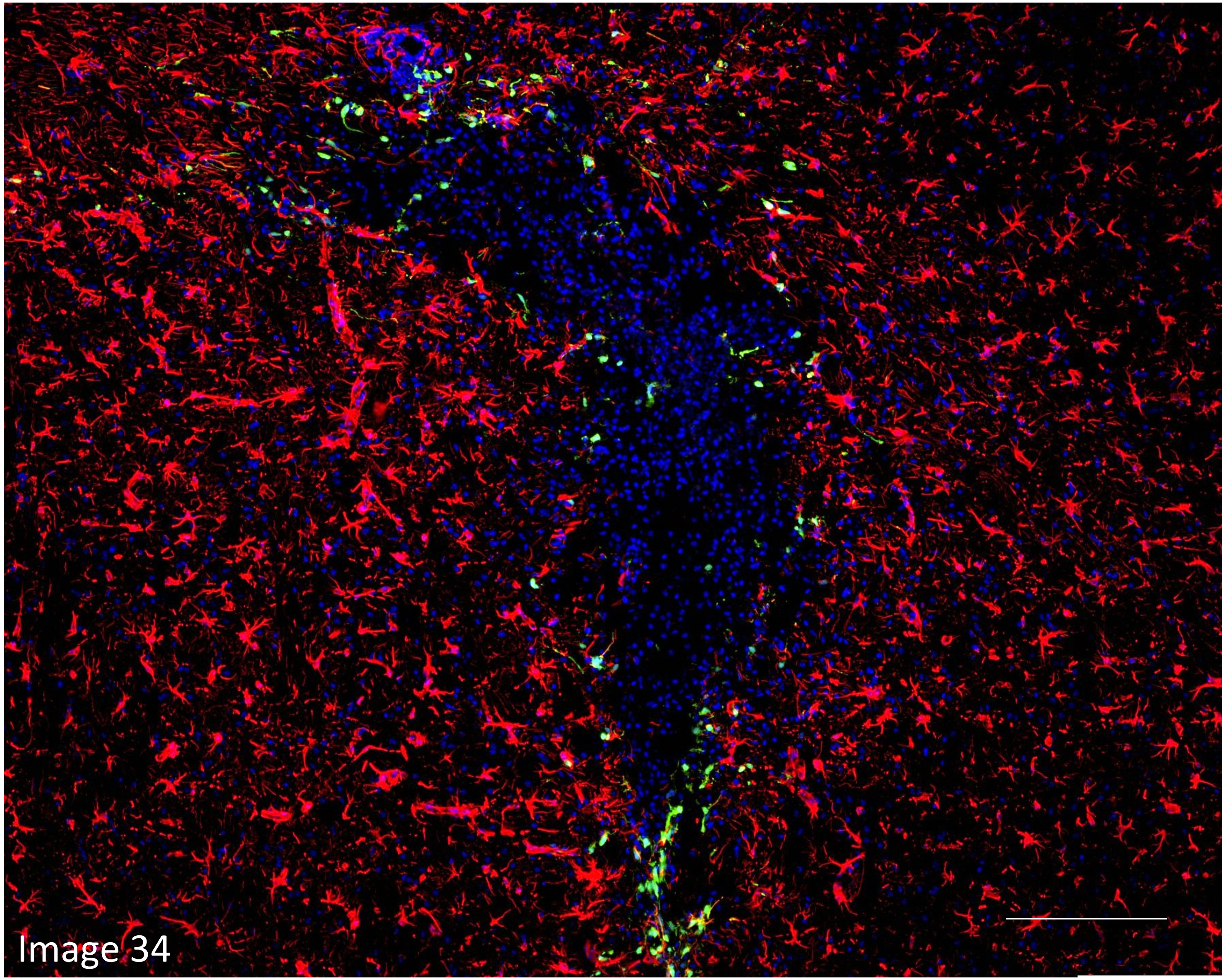

eGFP/TOPRO3/GFAP – day 7

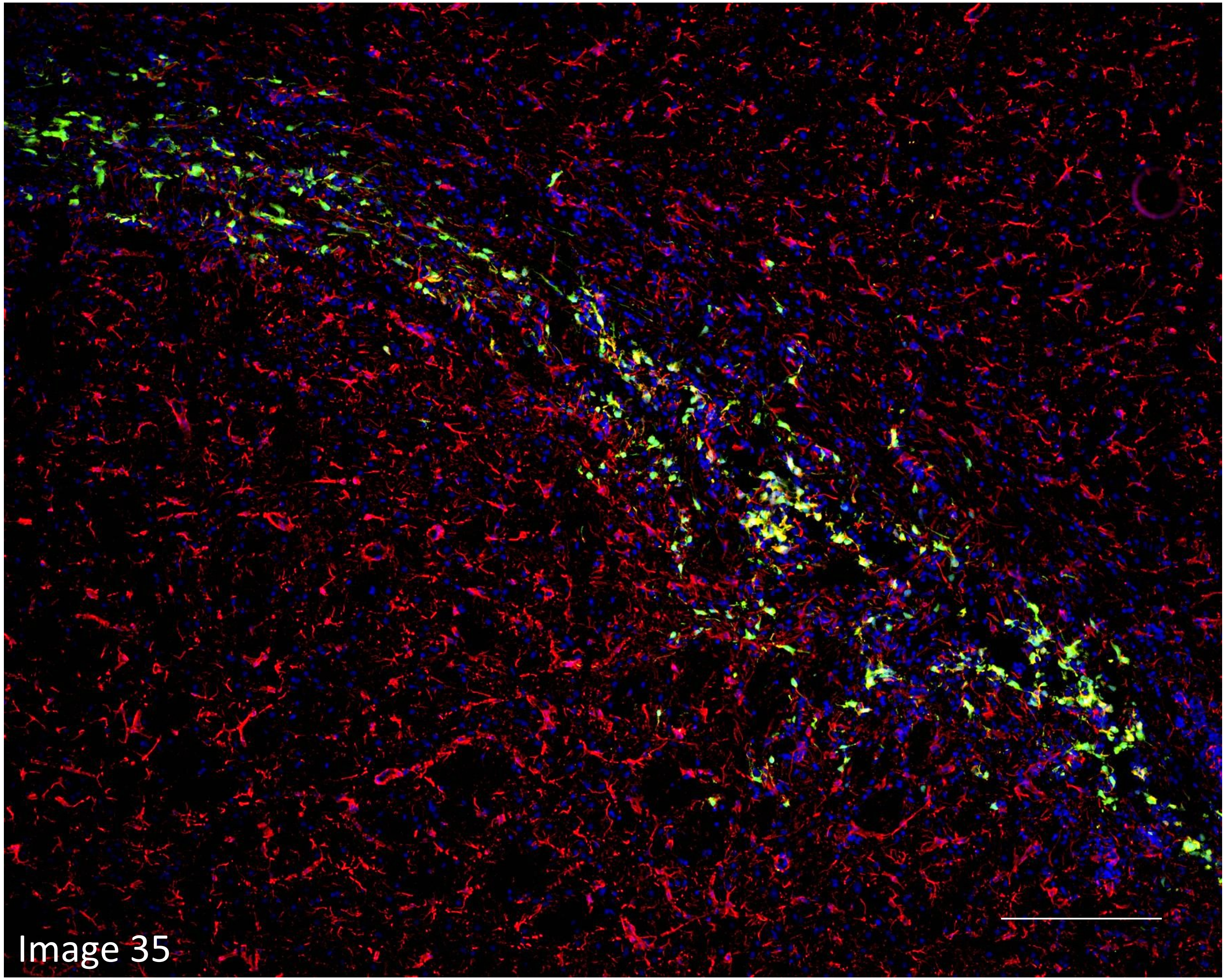

eGFP/TOPRO3/GFAP – day 14

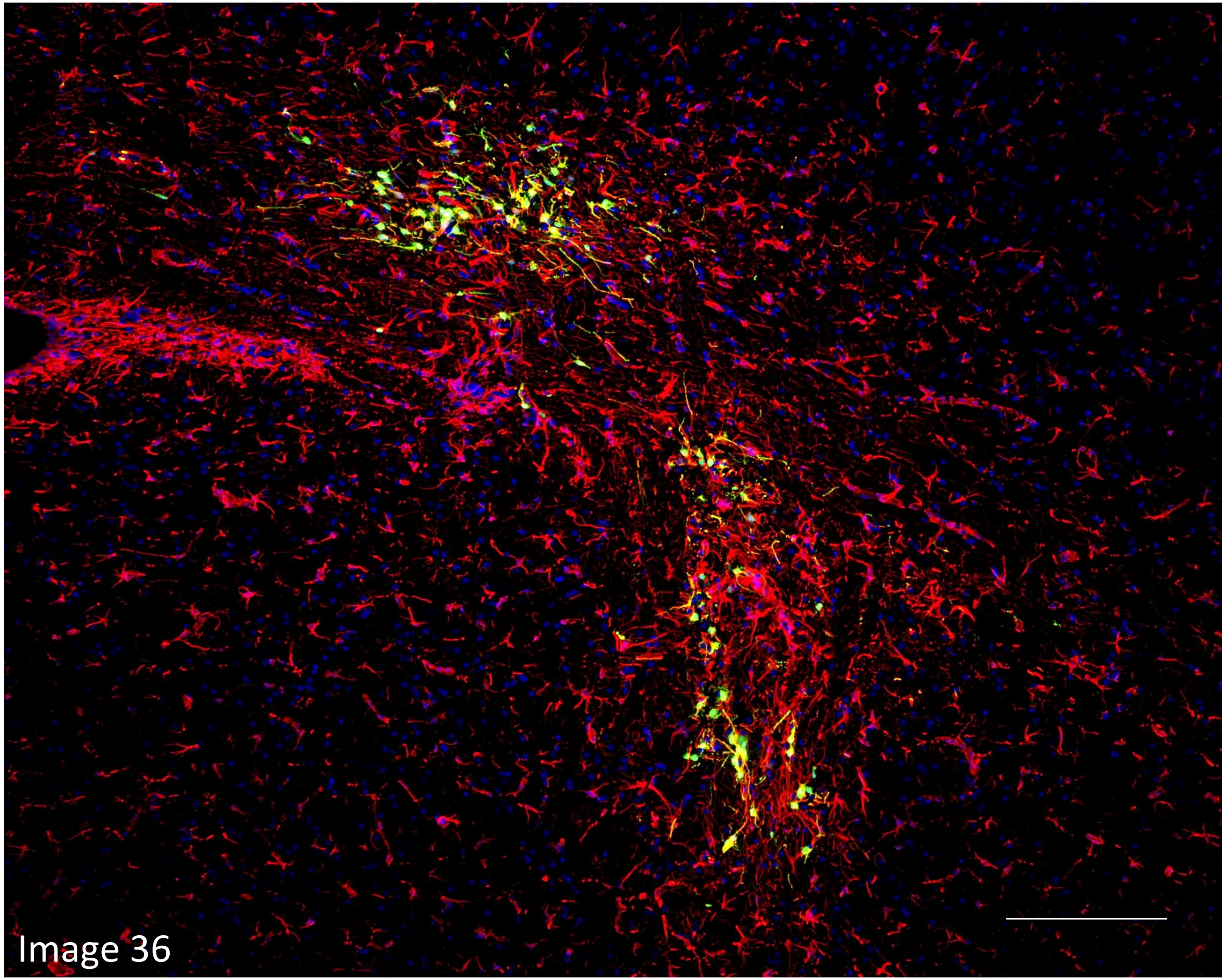

Figures S2:

Histological analysis of in vivo NSC graft apoptosis.

This section contains larger versions of the images presented in figure 4.

Direct eGFP fluorescence (in green) combined with TOPRO3 staining (false colour representation in blue) and combined with the control immunofluorescence staining for TUNEL (in red, CONTROL STAINING, Images 1&2) and the specific immunofluorescence staining for TUNEL+ apoptotic cells (in red, TUNEL STAINING, Images 3&4) at day 0 and day 1 post-implantation. Representative images were chosen from multiple mice analysed at each time point (n=2). The provided scale bars indicate 100µm.

eGFP/TOPRO3/CONTROL STAINING – day 0

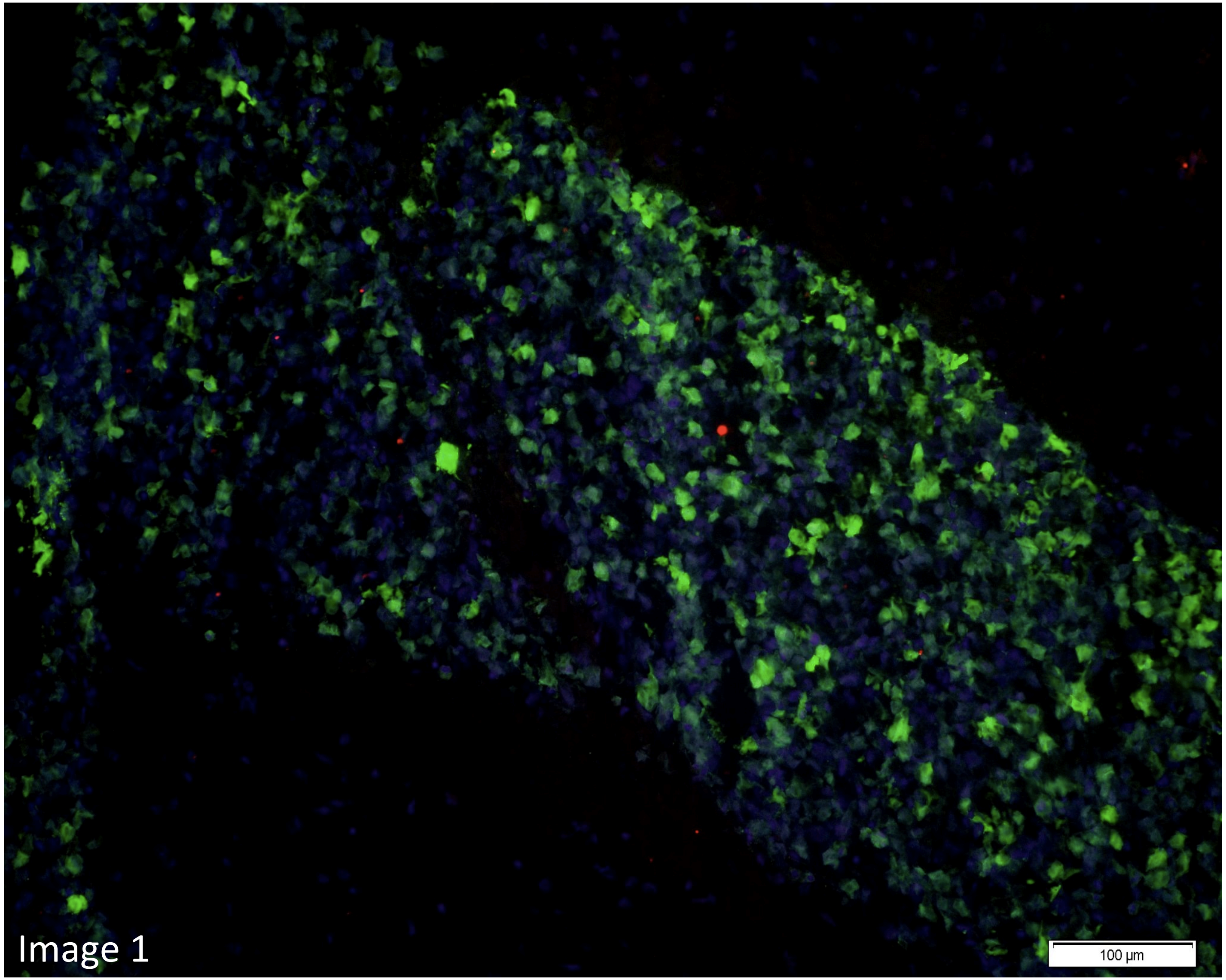

eGFP/TOPRO3/CONTROL STAINING – day 1

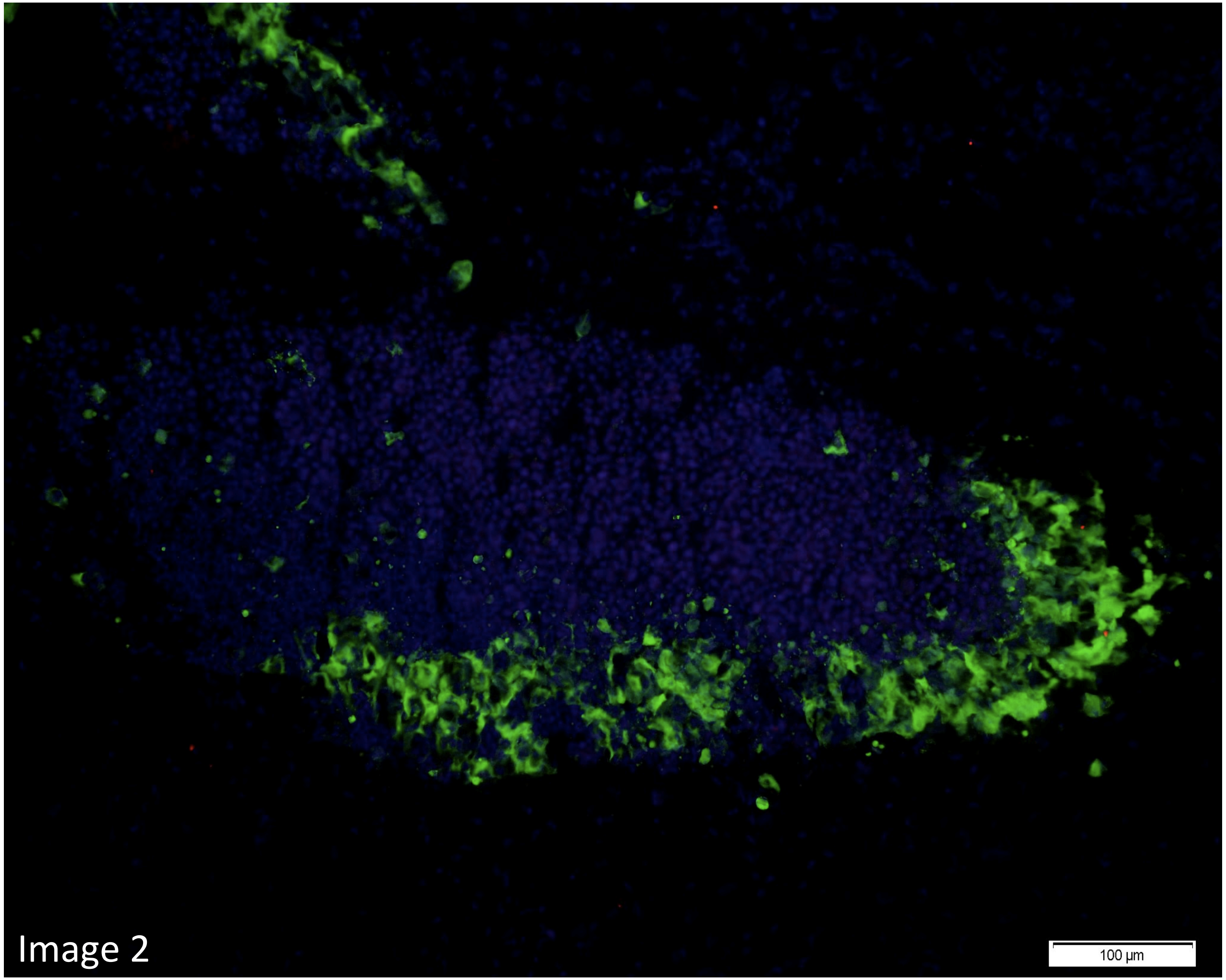

eGFP/TOPRO3/TUNEL STAINING – day 0

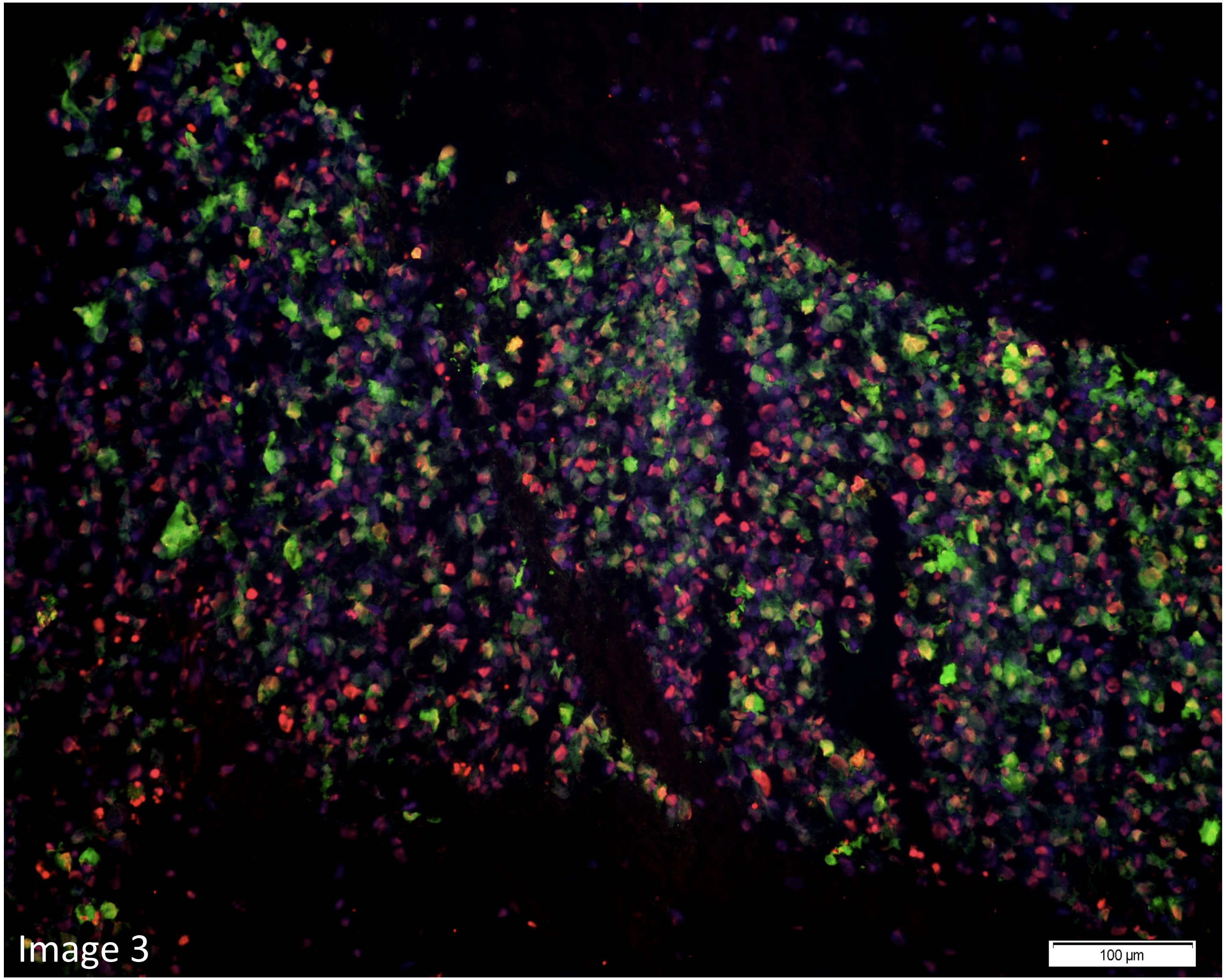

eGFP/TOPRO3/TUNEL STAINING – day 1

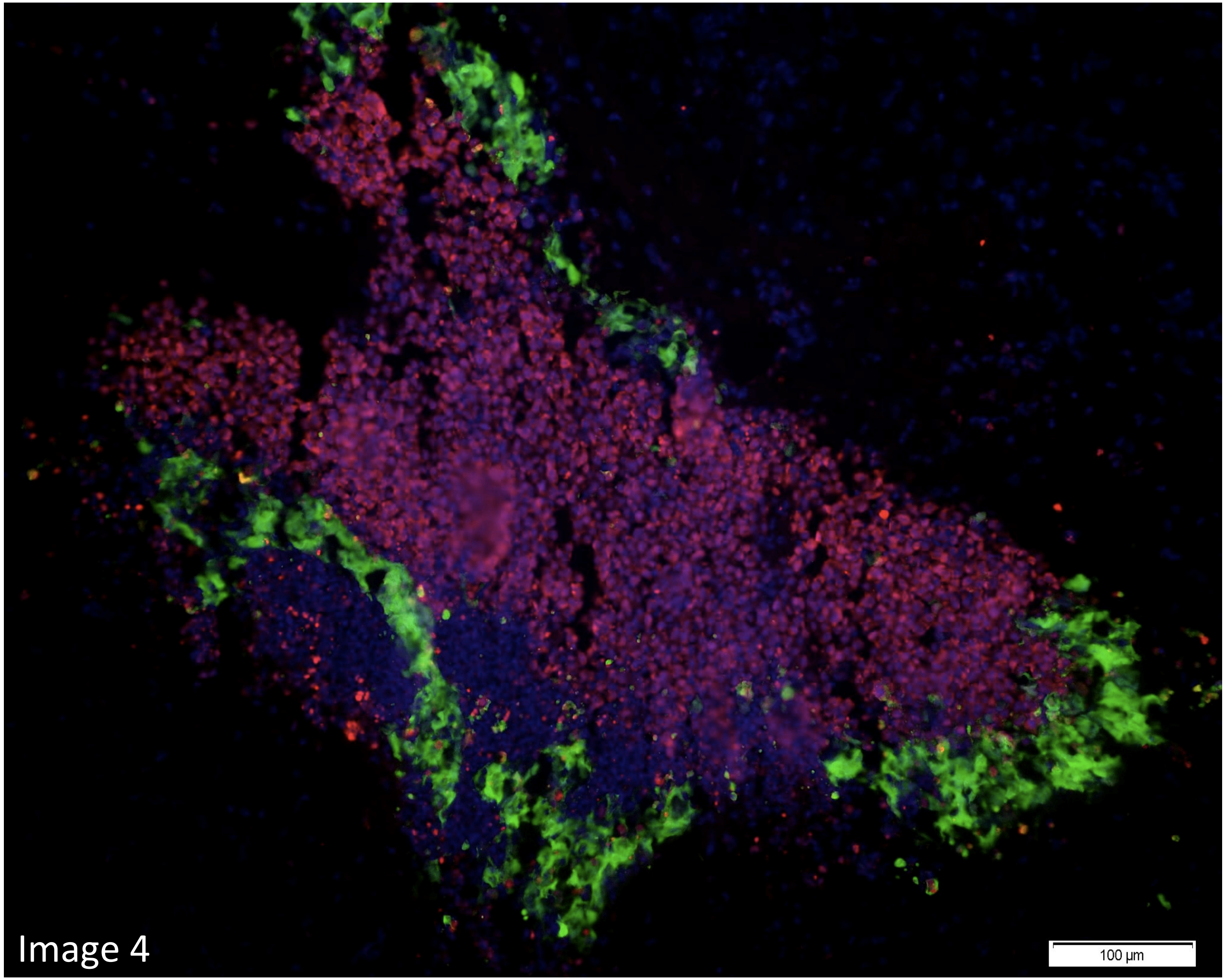

Supplement: Additional file 1 — Histological analysis of neural stem cell (NSC) graft survival, endogenous glial cell responses and in vivo NSC graft apoptosis. This file contains larger images of those presented in Figure 2 (Figure S1) and Figure 4 (Figure S2). [file scrt147-S1.PDF]
